# Parameterizing sequence alignment with an explicit evolutionary model

**DOI:** 10.1186/s12859-015-0832-5

**Published:** 2015-12-10

**Authors:** Elena Rivas, Sean R. Eddy

**Affiliations:** 1 Department of Molecular and Cellular Biology, Harvard University, Cambridge, 02138 MA USA; 2 Howard Hughes Medical Institute, 4000 Jones Bridge Rd, Chevy Chase, 20815 MD USA; 3 John A. Paulson School of Engineering and Applied Sciences, 16 Divinity Avenue, Cambridge, 02138 MA USA; 4 FAS Center for Systems Biology, Harvard University, 16 Divinity Avenue, Cambridge, 02138 MA USA

**Keywords:** Evolutionary models, Hidden Markov models, Insertions and deletions

## Abstract

**Background:**

Inference of sequence homology is inherently an evolutionary question, dependent upon evolutionary divergence. However, the insertion and deletion penalties in the most widely used methods for inferring homology by sequence alignment, including BLAST and profile hidden Markov models (profile HMMs), are not based on any explicitly time-dependent evolutionary model. Using one fixed score system (BLOSUM62 with some gap open/extend costs, for example) corresponds to making an unrealistic assumption that all sequence relationships have diverged by the same time. Adoption of explicit time-dependent evolutionary models for scoring insertions and deletions in sequence alignments has been hindered by algorithmic complexity and technical difficulty.

**Results:**

We identify and implement several probabilistic evolutionary models compatible with the affine-cost insertion/deletion model used in standard pairwise sequence alignment. Assuming an affine gap cost imposes important restrictions on the realism of the evolutionary models compatible with it, as single insertion events with geometrically distributed lengths do not result in geometrically distributed insert lengths at finite times. Nevertheless, we identify one evolutionary model compatible with symmetric pair HMMs that are the basis for Smith-Waterman pairwise alignment, and two evolutionary models compatible with standard profile-based alignment.

We test different aspects of the performance of these “optimized branch length” models, including alignment accuracy and homology coverage (discrimination of residues in a homologous region from nonhomologous flanking residues). We test on benchmarks of both global homologies (full length sequence homologs) and local homologies (homologous subsequences embedded in nonhomologous sequence).

**Conclusions:**

Contrary to our expectations, we find that for global homologies a single long branch parameterization suffices both for distant and close homologous relationships. In contrast, we do see an advantage in using explicit evolutionary models for local homologies. Optimal branch parameterization reduces a known artifact called “homologous overextension”, in which local alignments erroneously extend through flanking nonhomologous residues.

**Electronic supplementary material:**

The online version of this article (doi:10.1186/s12859-015-0832-5) contains supplementary material, which is available to authorized users.

## Background

Despite the apparent maturity of both the sequence similarity searching and phylogenetic inference fields, and despite the fact that inference of sequence homology via sequence alignment is itself obviously an evolutionary question, standard sequence comparison methods for homology search such as BLAST [1] or profile HMMs [2] still do not depend on an explicitly divergence-dependent evolutionary model for their parameterization. Instead standard sequence alignment methods use parameterizations that assume fixed evolutionary divergence times either implicitly or explicitly. The basis for deriving standard log-odds substitution scores from a rate-dependent continuous time Markov model of the substitution process is straightforward and well known [3]. The difficulty has been in reconciling the standard affine gap penalties used in fast, efficient sequence alignment and database search methods with a satisfactory continuous time evolutionary model of the insertion and deletion process.

Imposing an evolutionary model in standard affine pair and profile HMMs would allow us to optimize the parameterization (branch length) to the apparent relatedness of each comparison, instead of assuming that all sequences are at the same evolutionary distance from each other. We expect to see a gain from such parameter optimization. For example, one advantage of optimizing score systems for evolutionary distance has been shown when choosing substitution matrices for scoring ungapped alignments of different lengths [4]. Using a *divergent* scoring system, a long divergent homologous region may be detected by summing many weak positive scores, whereas a short conserved homologous region may not accumulate enough total positive score to rise to significance. On the other hand, using a *conserved* scoring system, a short conserved homologous region may be detected because conserved residues received higher positive score per position, whereas a long divergent homologous region may go unrecognized because mismatches are heavily penalized. An optimal scoring system can find the best compromise given the length of the compared sequences and their relatedness.

A large body of work has established the feasibility of evolutionary models of insertion and deletion processes. Several models have been proposed including the well-known TKF91 and TKF92 models [5, 6], some precursor models [7, 8], and other related models [9, 10]; tree HMMs [11–13]; models that treat gaps as an extra residue [14, 15]; pair HMMs implementing an approximate evolutionary model [16]; and other more complex but analytically intractable models [17, 18]. The desire to synchronize the evolutionary distance of a given substitution matrix to that of the indel parameters has been recognized, and sets of score parameters (including gap costs) at different discrete evolutionary distances have been proposed [19]. Nevertheless, standard sequence comparison methods such as BLAST [1] or SSEARCH [20] that implement a standard three-state (Match/Insert/Delete) affine gap cost recursion (with symmetric treatment of the Insert/Delete states) do not use explicit evolutionary models that would automatically set the parameters to a variable divergence time.

We are most interested in assessing the value of evolutionary models for profile HMMs. Profile HMMs can be understood as a generalization of sequence/sequence comparison methods to a sequence/profile comparison in which the profile compiles information about one sequence or a collection of homologous sequences. Groups of sequences can be efficiently aligned to the profile avoiding a costly all-to-all comparison. Profile HMM methods are used extensively for protein and DNA homology analysis [21–24]. Standard profile HMM packages (like other standard sequence similarity methods) do not explicitly utilize any evolutionary model [25, 26].

Profile HMMs are usually parameterized “long branched” to optimize for remote homology detection. Thus, the advantages of an optimal-branch model would be in situations where having a higher score per conserved position is advantageous. Such situations include: (1) to reduce alignment overextension artifacts in the case of homologous sequences embedded in nonhomologous sequence [27, 28]; (2) to improve the identification of short homologies (such as in metagenomic reads); (3) to improve alignment accuracy by better discriminating mutations from indels, avoiding the artifact of indel collapse, in which two independent insertions are aligned together as if they were homologous [29].

In this paper, first we investigate the constraints imposed by the affine-gap architecture in probabilistic evolutionary models. In addition to the TKF91 and TKF92 models [5, 6], we identify new affine evolutionary models that have richer parameterizations with more variables. Importantly (and beyond what the TKF models can do), we identify one evolutionary model compatible with standard pair HMMs with a symmetric treatment of insertions and deletions (as in the Smith-Waterman algorithm), and two evolutionary models compatible with profile HMMs.

We have implemented these affine evolutionary models (including TKF91 and TKF92) into a pair HMM-based probabilistic local alignment program called e2msa. The e2msa method provides a platform for the level comparison of different affine evolutionary models.

We use e2msa to test the effect of optimizing branch lengths on alignment accuracy and homology coverage (fraction of the true homology incorporated into the alignment). We produce a benchmark of global (full-length) homologies. To test for nonhomologous overextension artifacts, we produce a second alignment benchmark of local homologies embedded into nonhomologous sequences.

## Results and discussion

### Constraints on evolutionary models compatible with affine gap cost

The frequency of insertions and deletions (indels) in biological sequences deviates significantly from the geometric length distribution implicit in any affine gap cost model [30, 31]. However, affine gap cost has become standard because it is a good compromise between realism and computational efficiency. Here, we have tried to elucidate what kind of evolutionary model is compatible with well-established computationally efficient comparative models, such as pair HMMs [3] or profile HMMs [2, 3, 25, 32].

An evolutionary model starts by proposing a *microscopic model* that dictates the rules describing how inserted residues appear or disappear instantaneously, as well as how ancestral residues can mutate or disappear in one single event. The microscopic model’s repertoire of single events are described in terms of some constant parameters, the so-called rates. Solving the differential equations derived from the microscopic model results in a *macroscopic model* described in terms of time-dependent conditional probabilities (or log-odds scores derived from those) which become functions depending on the rates and a divergence time parameter t. The macroscopic model describes the ancestral/descendant sequence relationship after millions of years of evolution, while the microscopic model describes the same relationship only after very short times.

Throughout this paper, the word “**insert**” has a special meaning (as opposed to the terms insertion or inserted residues). “Insert” specifically stands for the collection of all inserted residues in between any two ancestral positions (at a given time), regardless of whether the flanking ancestral residues are alive or not at the time. This concept arises naturally in the framework of an evolutionary profile HMM where each position in the profile represents an ancestral residue, such that in between any two profile positions an arbitrary number of residues (an insert) can exist. In an evolutionary profile HMM, residues in a given insert have been generated using the same rate parameters (possibly at different times), and different inserts use different rates.

In a macroscopic affine model, the cost of an insert of length n is a + bn, consisting of the cost of opening (a) and the cost of extending the insert (b) as two independent parameters. When the gap open cost is set to zero, it is called a linear model. Affine models fit biological data better than linear models, by making it more costly to start an indel than to extend it. For instance, NCBIBLAST typically uses a default gap-open cost of -11, and a gap-extend cost of -1; in PHMMER [21], the gap-open probability is 0.03, while the gap-extend probability is 0.40.

The seminal TKF91 evolutionary model assumes a microscopic model in which inserted residues are all added (or removed) identically to each other and one at a time (with rate *λ* for insertions and *μ* for deletions). As a result, the macroscopic model for TKF91 is essentially a linear gap cost model^1^. In addition, because TKF91 is meant for sequence/sequence comparisons, it assumes that when an ancestral residue is deleted it does not leave any trace behind. In a sequence/profile comparison scenario, the profile plays the role of the ancestor, and even if an ancestral residue might have died at some point, inserted residues associated to that profile position retain their own position-specific parameterization. Thus, when using position-specific scores in profile evolutionary models, it is not entirely unreasonable to maintain at all times a “memory” of all the consensus (ancestral) positions in the profile.

In an attempt to make affine evolutionary models, one might imagine making two modifications (in addition to the memory effect) to the *microscopic model* of TKF91. One modification is to allow residues to appear and disappear not one at a time but in groups according to a geometric distribution (with probability (1−*v*_*I*_)(*v*_*I*_)^*n*−1^ and (1−*v*_*D*_)(*v*_*D*_)^*n*−1^ for *n* appearing or disappearing residues respectively). The second modification is to allow the rates at which these single-event group insertions appear to be different whether they open a new insert (with rate *λ*) or just expand an existing insert (with rate *λ*_*I*_), and similarly for the deletion of inserted residues, the model distinguishes whether the insert disappears completely (with rate *μ*) or it just shrinks (with rate *μ*_*I*_). Because of the geometric nature of the single events allowed for insertions (addition and removal of groups of them), we refer to this model as the Geometric (GM) model.

Unfortunately, the GM model does not have an affine macroscopic solution. Allowing elementary events with geometric length distributions does not result in geometrically distributed insert lengths. In the Additional file 1, we present an explicit description of the GM model, as well as a proof that the macroscopic GM model cannot have a geometric form. The result comes from proposing a geometric distribution as an ansatz for solving the differential equations of the GM model, and showing that a solution cannot be reached.

**Fig. 1:**
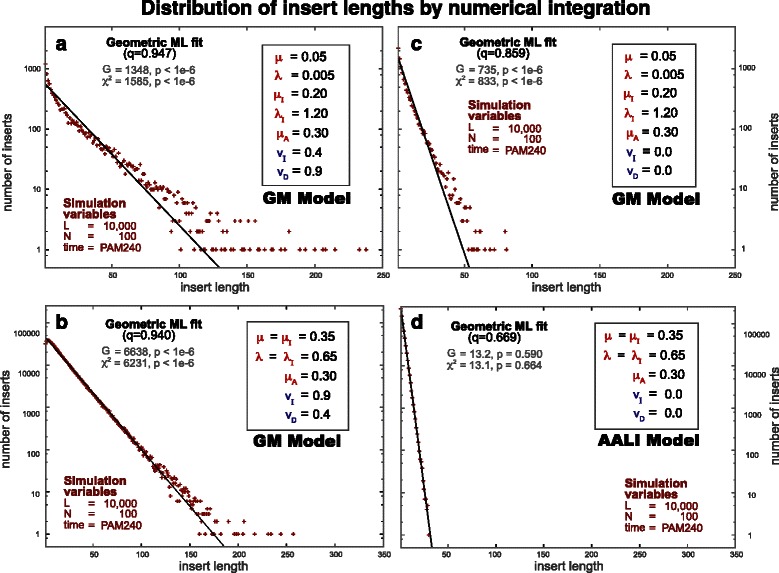
Under the GM evolutionary model, the length of inserts does not follow in general a geometric distribution, and therefore this model is incompatible with affine gap cost alignment. A sample of N=100 ancestral sequences of length L = 10,000 are evolved according to the GM model to different divergence times. The y-axis is given in logarithmic scale, thus a geometric distribution becomes a straight line. At a given divergence time t, evolved sequences are obtained by sampling from the infinitesimal time microscopic model at discrete intervals of *δ* *t*=10^−5^. For the particular divergence time corresponding to PAM240 (*t*=2.2), we present the histogram of insert lengths (*i.e.* the number of residues between any two ancestral positions) for several sets of parameter values. The black line corresponds to a maximum likelihood fit of the data to a geometric distribution of the form *q* ^*l*^(1−*q*) with its corresponding G and *χ* ^2^ goodness-of-fit tests and their corresponding probabilities. Panels (**a**) and (**b**) both consider cases in which residues are added according to geometric distributions. In particular, panel (**b**) considers that case in which *λ*=*λ*_*I*_ and *μ*=*μ*_*I*_. In panels (**c**) and (**d**) all geometric parameters are zero, and residues are added one at a time. The particular parameters in Panel (**d**) corresponds to the AALI evolutionary model, a special case of the GM model in which insert length fits a geometric distribution. Notice that a straight line (geometric fit) is not sufficient to demonstrate affine models, because linear models (like the AALI model) also produce geometric insert lengths

In addition, in Fig. 1 we confirm by numerical integration the non-geometric nature of the macroscopic GM model with several examples (see Fig. 1 a). The macroscopic model is not geometric even when the open/extend rates for insertions and deletions are identical to each other (*λ*=*λ*_*I*_ and *μ*=*μ*_*I*_) as shown in Fig. 1 b. Furthermore, even without geometrically distributed elementary events (*v*_*I*_=*v*_*D*_=0), assuming that the rate of an emerging/disappearing insert is different from that of an expanding/contracting insert (*λ*≠*λ*_*I*_ and *μ*≠*μ*_*I*_) still falls outside affine gap cost models, as shown in Fig. 1 c.

**Fig. 2:**
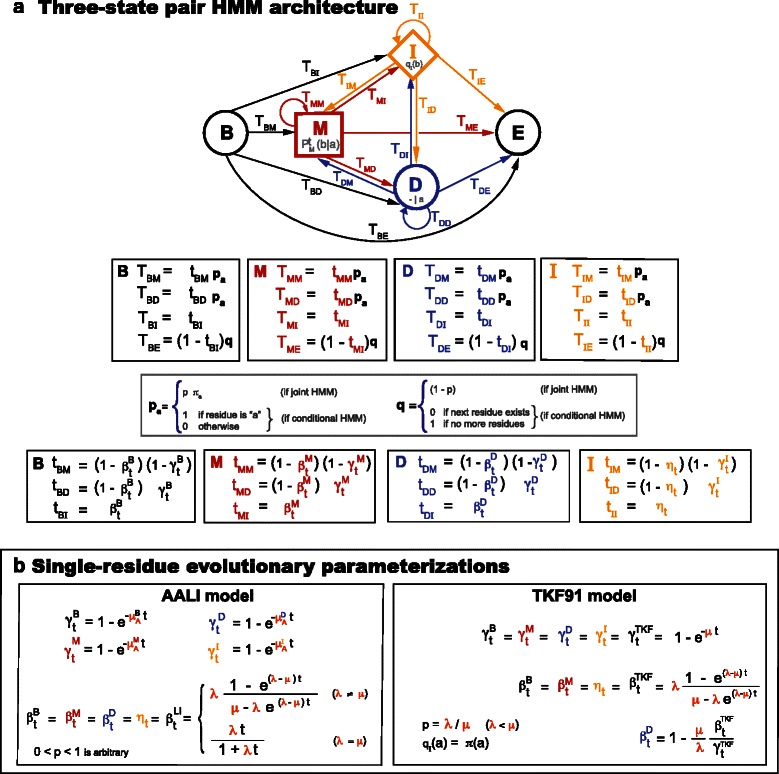
Standard affine three-state pair HMM with two explicit evolutionary parameterizations. **a** Standard probabilistic three-state M/D/I HMM (plus the customary B and E states) to describe homologous sequences under an affine gap cost. We simultaneously describe the joint and conditional versions of the HMM. For the joint version, we assume one of the sequences is generated with a length according to a geometric distribution of Bernoulli parameter *p*, and residues drawn from a probability distribution *π*. Joint residue match emissions occur with probability *P*_*t*_(*a,b*)=*π*(*a*)*P*_*t*_(*b*∣*a*), and conditional match emissions occur with probability *P*_*t*_(*b*∣*a*). Inserted residues follow a probability distribution *q*_*I*_ which in principle could be different from *π*. The transition parameters t_XM_,t_XD_,t_XI_ are probabilities valued between zero and one. The HMM is normalized in its two versions (joint or conditional) as long as t_XM_+t_XD_+t_XI_=1 for all states *X*={*B,M,D,I*}. **b** We write the transition probabilities of the HMM in terms of several elementary probability functions *γ* ^{*B,M,D,I*}^(*t*) (the probability of deleting an ancestral residue), *β* ^{*B,M,D,I*}^(*t*) (the probability of opening an insert), and *η*(*t*) (the probability of extending an insert). The HMM transition probabilities are automatically normalized for arbitrary elementary probability functions. Here we provide explicit time-dependent descriptions of the probabilistic elementary functions of the pair HMM according to the AALI and the TKF91 evolutionary models

It is only in the absence of both modifications (but maintaining the memory effect) that we obtain geometric distributions of insert lengths (Fig. 1 d). However, the resulting insert distribution is linear, not affine. This linear for insertions model can be made affine for the deletion of ancestral residues by assuming that a different rate is used depending on whether the deletion happens after an ancestral substitution, an ancestral deletion, or after an inserted residue. We name this model the affine ancestral and linear inserts (AALI) model. The AALI model can be solved analytically. Details of the differential equations and solutions of the AALI model are given in the Additional file 1. In Fig. 2, we provide as a summary a state representation of the macroscopic AALI model as a standard three-state pair HMM. The AALI model is not constrained by reversibility, which allows some richness of parameters. A particular tying of the AALI parameters produces a reversible model, which we refer to as the Linear Reversible (LR) model. Details of the LR model are given in the Additional file 1.

The AALI model (more specifically, the LR model) is comparable to the TKF91 model since both assume at the microscopic level that residues are added/deleted one at a time. The two models have an important difference: the AALI microscopic model, influenced by the fact that it aims at parameterizing position-specific models, maintains a memory throughout the evolutionary process of all ancestral positions even after their death. In the TKF91 model on the other hand, because it assumes a non-position-specific model, inserted residues after a deleted ancestral residue are collapsed with any other previous inserted residues associated to a different ancestral residue. This one microscopic difference results in some important macroscopic differences between the two models. The macroscopic TKF91 model also admits a three-state HMM representation [9, 33], as shown in Fig. 2. We present a comparison between the LR model and the TKF91 model in the Additional file 1.

#### Affine evolutionary models by fragments

**Fig. 3:**
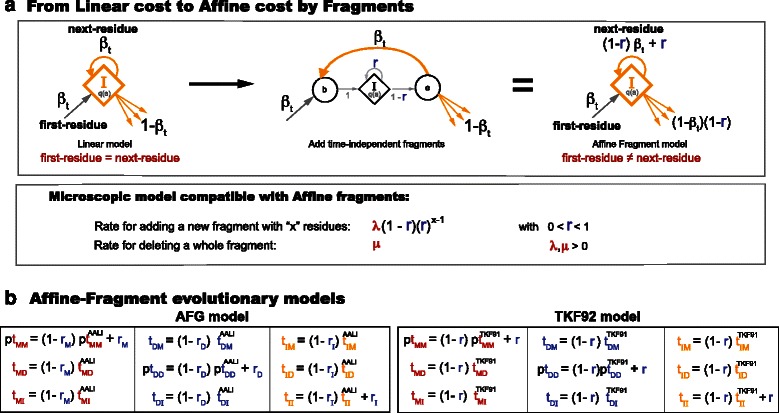
Affine fragment evolutionary models. **a** It is possible to convert a linear-cost model into an affine-cost one by adding units of indivisible “fragments”, each having a geometrically-distributed length. The resulting macroscopic affine model is compatible with a microscopic evolutionary model in which groups of residues are added/removed together instantaneously with a length controlled by a time-independent geometric Bernoulli parameter, with the condition that groups of residues created instantaneously together have to also die together. **b** Macroscopic fragments can be added to any state with a self-loop transition. The AFG model (Table 1) is a fragment derivative of the AALI model. In the TKF92 model (a fragment derivative of TKF91) fragments are added to all three M/D/I states with the same parameter *r*. The AFG model becomes reversible in the particular case that it uses the transition probabilities of the LR model, and by setting the Insert and Delete fragment parameters equal to each other (*r*_*I*_=*r*_*D*_). We call the reversible version of the AFG model the affine fragment reversible (AFR) model

The AALI model, though it is only linear for insertions, can be used as a starting point to generate affine variants using the “fragments” technique introduced with the TKF92 evolutionary model (a fragment-derivative of TKF91) [6]. From a macroscopic perspective, a linear insert state can be made affine by replacing the emitting self-looping state with another geometric state machine (adding a new geometric parameter *r*). The modified HMM can be converted back to the original state machine, but now with an affine transition probability (depending on the added geometric parameter) as described in Fig. 3 a. The concept “fragment” was coined with the introduction of the TKF92 model because the microscopic interpretation of this procedure requires assuming that all the residues created instantaneously by the added state machine have to be removed instantaneously as they were created without further subdivisions. The affine for insertions fragment-derivative of our AALI model is named the AFG model (see Fig. 3 b). Because we do not insist on reversibility, the AFG model has the freedom to use independent fragment parameters for the different states (Match, Delete or Insert).

**Table 1:** Evolutionary models compatible with pair and profile HMMs

Microscopic model				Macroscopic model	
Evolutionary model	Total # free parameters	Rates	Geometric parameters	# States minimal pair HMM	Other properties
*Single-residue models*(Fig. 2, Additional file 1: Figure S1 and S2)					
aali	6	$\lambda,\mu,\mu _{A}^{\{\text {\textit {M,D,I}}\}}$	*p*	3	affine ancestral residues; linear inserts
li	4	*λ*,*μ*,*μ*_*A*_	*p*	1	linear; particular case of aali
lr	2	*λ*,*μ*_*A*_,(*μ*=*λ*+*μ*_*A*_)	(*p* ^lr^=*λ*/*μ*_*A*_)	1	reversible; particular case of li
tkf91	2	*λ*,*μ*	(*p* ^tkf^=*λ*/*μ*)	2	quasi linear; [5]
*Fragment models*(Figs. 3 and 4)					
afg	9	$\lambda,\mu,\mu _{A}^{\{\text {\textit {M,D,I}}\}}$	*r*_*M*_,*r*_*D*_,*r*_*I*_,*p*	3	fragment-derivative of aali
afr	4	*λ*,*μ*_*A*_,(*μ*=*λ*+*μ*_*A*_)	*r*_*M*_,*r*_*D*_=*r*_*I*_,(*p* ^lr^)	3	reversible; compatible with Smith-Waterman
tkf92	3	*λ*,*μ*	*r*, (*p* ^tkf92^)	3	fragment-derivative of tkf91; [6]
*Compatible with profile HMMs*(Fig. 5)					
aif	7	$\lambda,\mu,\mu _{A}^{\{\text {\textit {M,D,I}}\}}$	*r*_*I*_,*p*	3	inserts-only fragment-derivative of aali
aga	7	$\lambda,\mu,\mu _{A}^{\{\text {\textit {M,D,I}}\}}$	*s*_*I*_,*p*	3	time-independent geometric inserts

*From a microscopic perspective* we have three types of models: (1) single-residue models in which residues are inserted and deleted instantaneously one at a time; (2) fragment models that can insert/delete/replace several residues at the time, but where residues created simultaneously act as an indivisible unit (thus the name fragments); (3) The aga model where in one single event inserts can appear or disappear, but they cannot grow or shrink. The aali model (and its particular cases the li and lr models) as well as the tkf91 model belong to the first category of single-residue models. Fragment models can be built starting from any of the single-residue models. In the aga model, the distribution of inserts length is geometric but it does not change with time. *From a macroscopic perspective*, the li model (and its particular case the reversible lr model) fit into a one-state linear HMM, but the similar model tkf91 requires at least a two-state HMM. The aali model requires a three-state HMM because it is affine with respect to the fate of ancestral residues. The number of states of the minimal HMM does not include the customary begin (B) and end (E) states; we assume in all cases that $\mu _{A}^{\textsc {b}}=\mu _{A}^{\textsc {m}}$. All fragment models fit into a standard three-state HMM. Parameters that are not independent are given in parentheses. The distribution of ancestral sequences for the fragment model tkf92 is approximately a geometric distribution: the expression of *p* ^tkf92^ in terms of the free parameters of the model can be found in [6]. The affine fragment reversible (afr) model is a particular case of the afg model obtained as a fragment derivative of the lr model such that in order to preserve reversibility deleted and inserted fragments are drawn from the same geometric distribution (*r*_*D*_=*r*_*I*_). In the afr model insertions and deletions have identical treatment. There are two models compatible with profile HMMs of Krogh’s form [32]: the aif model and the aga model. The aif model is a particular case of the afg fragment model (with *r*_*M*_=*r*_*D*_=0). The aga model assumes the simplification that inserts are geometrically distributed with a time-independent (but position specific) Bernoulli parameter. All evolutionary models have been implemented in the alignment program e2msa

#### An evolutionary model (AFR) compatible with the Smith-Waterman algorithm

Sequence to sequence comparison methods based on the Smith-Waterman algorithm [34] such as BLAST or SSEARCH have a symmetric treatment of insertions and deletions. Finding an evolutionary parameterization of a Smith-Waterman-based comparative method requires having an affine gap cost and a reversible model.

**Fig. 4:**
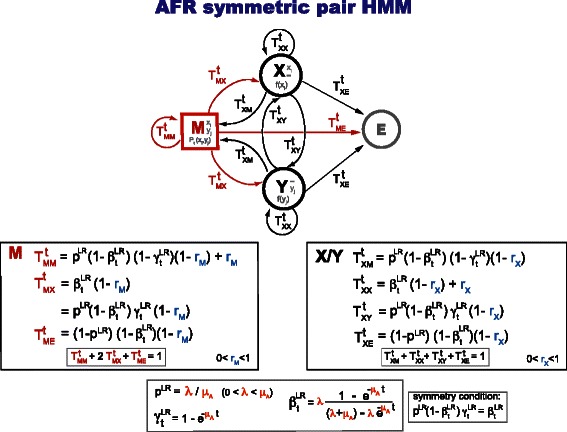
A symmetric evolutionary pair HMM. State machine representation of the AFR evolutionary model in joint form, where the X/Y state symmetry (reversibility) is obvious. The AFR model is a fragment derivative of the Linear Reversible (LR) model. We use capital letters for the transition probabilities in order to remember that these are joint probabilities. The normalization of the model may not be apparent from this representation. One should remember the reversibility condition of the LR model, given by $\beta _{t}^{\textsc {lr}} = p^{\textsc {lr}}(1-\beta _{t}^{\textsc {lr}})\gamma _{t}^{\textsc {lr}}$

There is a fragment derivative of the Linear Reversible (LR) model that is also reversible, thus compatible with the insertion/deletion symmetry required by the Smith-Waterman algorithm. We call this model the Affine Fragment Reversible (AFR) evolutionary model. To preserve the insertion/deletion symmetry, the AFR model must use the same fragment parameter for insertions and for deletions (*r*_*I*_=*r*_*D*_ in Table 1). See Fig. 4 for details about the AFR model. The AFR evolutionary model is described in the Additional file 1.

In the TKF models, the joint transition probabilities *M*→*I* and *M*→*D* are different from each other, and the joint transition probabilities *I*→*I* and *D*→*D* are also different from each other, as can be seen by inspection of Figs. 2 and 3. Thus, the TKF models cannot be used to parameterize symmetric three-state pair HMMs.

#### Two evolutionary models (AIF and AGA) compatible with profile HMMs

**Fig. 5:**
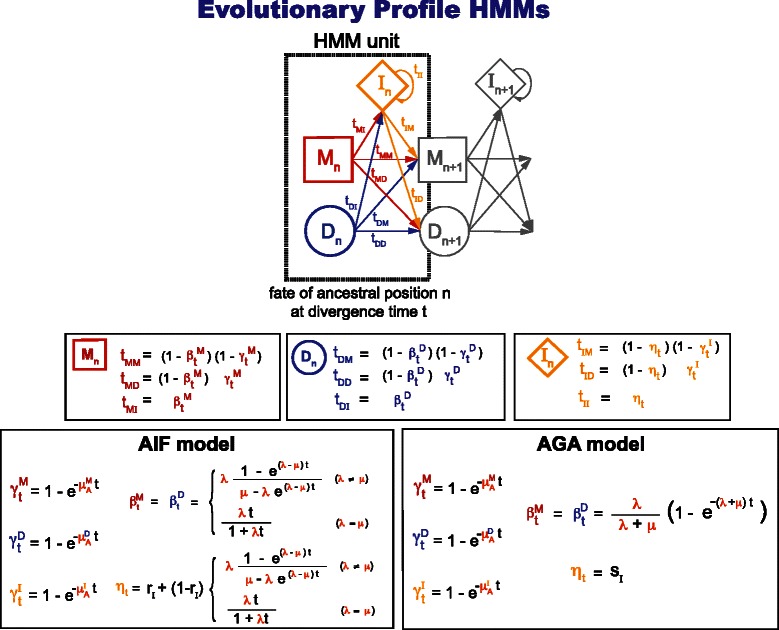
Two evolutionary description of a profile HMM. Notice that the AIF and AGA evolutionary models are not compatible with HMMER profile HMMs, which do not allow transitions from Delete to Insert and Insert to Delete

The fragment models AFG and TKF92 are not compatible with profile HMMs. The AFG and TKF92 models add fragments to all three self-looping states in the pair HMM, but in a profile HMM only the Insert is a self-looping state. We can create a variant of the AFG model in which fragments are only added to the Insert state, which we call the Affine Insert Fragment (AIF) model. (A similar variant cannot be made for TKF92, since all fragments are tied to use the same Bernoulli parameter.) The AIF evolutionary model is affine both for insertions and deletions, although for different reasons. Affine insertions occur because of the fragments, affine deletions occur because of the position-specific deletion rate constants. The AIF evolutionary model is also not reversible, and compatible with standard profile HMMs (see Fig. 5).

There is an alternative to the fragment method used by the AIF model in order to introduce evolutionary models for profile HMMs. It requires assuming that the length distribution of inserts does not change with time. We call this model the AGA model. (A pair HMM with time-independent insert length distribution has been introduced before [16]). Despite the simplification, we later verify that the AGA model performs comparably to the AIF model. The AIF and the AGA state machine representations are given in Fig. 5, and details about the models are given in the Additional file 1.

The AIF and the AGA models are compatible with profile HMMs that contain *D*→*I* and *I*→*D* transitions, including the original profile HMMs proposed by Krogh et al. [32]. The HMMER HMM profile software uses a modified “plan 7” architecture that disallows *D*→*I* and *I*→*D* transitions [21]. It seems quite forced to propose an evolutionary model that would not allow one to find inserted residues after deleted ancestral residues. The HMMER architecture would need to be revisited to take advantage of the AIF and AGA models.

In summary, we report that: (1) In general, creating evolutionary models compatible with affine-cost alignment is not easy; geometrically distributed microscopic insertions do not yield geometrically distributed macroscopic inserts. (2) There is an affine gap-cost model (the AFR model) compatible with the insertion/deletion symmetry assumptions of Smith-Waterman-based comparative methods. (3) There are two ways of obtaining the affine costs of profile HMMs, either by adding fragments to the Insert state (the AIF model), or by assuming that inserts are an indivisible unit that follow a time-independent geometric length distribution (the AGA model).

A compilation of affine probabilistic evolutionary models with analytic solutions identified by us and others and their properties is given in Table 1.

### A time-dependent parameterization for BLAST

A typical parameterization of BLAST is to use the BLOSUM62 substitution scoring matrix, together with an open cost of -11 and a extend cost of -1 (all given in 1/2 bit units), which has been obtained after years of empirical determination by trial and error [35]. Alternative sets of affine gap cost parameters appropriate for different substitution matrices modeling different degrees of sequence similarity have been obtained empirically [36]. Here we propose a time dependent parameterization of BLAST (or any other sequence to sequence comparison method based on the Smith-Waterman algorithm) based on the AFR evolutionary model. We also propose a method to estimate the evolutionary parameters based on the requirement that it yields the (–11/-1) particular values at one particular time point.

We have shown that the affine fragment reversible (AFR) model is compatible with the particular insertion/deletion symmetry implied in Smith-Waterman-type comparisons. From an evolutionary perspective, we could assume that a set of specific parameter values (such as the above mentioned -11/-1) corresponds to the parameter values under the AFR evolutionary model at one specific (and arbitrary) fixed-time point (*t* ^⋆^). Then, we will show that calculating the probabilities at any other time becomes an algebraic problem of solving for the rates and Bernoulli parameters of the evolutionary model given particular fixed time values of the probabilities. (We have taken a similar approach elsewhere [37]).

Smith-Waterman’s “open+extend” cost is the score of the first residue in an indel, and the “extend” cost is the score of any other residue in the indel. Based on the probabilistic symmetric AFR pair HMM (Fig. 4), we propose to use

$$\begin{array}{@{}rcl@{}} \text{extend} &=& \log\mathrm{T}_{\text{XX}},\\ \text{open} + \text{extend} &=& \log\mathrm{T}_{\text{MX}} + \log(1-\mathrm{T}_{\text{XX}}), \end{array} $$

where in the open score in addition to the cost of entering an indel (logT_MX_), we need to include the cost associated with exiting the indel state which has no counterpart in the affine gap cost Smith-Waterman scheme. This particular mapping is an approximation.

In fact, it can only be an approximation. In Smith-Waterman, all indels are scored equally independently of their context. In its symmetric pair HMM counterpart, indels have different scores (log probabilities) depending on where they occur (flanked by conserved regions and/or indels in the other sequence). In the probabilistic Smith-Waterman, it is hard to make the different possible costs equal to each other. From an evolutionary perspective, one does not want to set them identical to each other. For more conserved parameterizations, one would expect to have more indels flanked by homologous residues. For more divergent parameterizations, one would expect see more instances of indels flanked by indels. The term log(1−T_XX_) includes both contributions ($\mathrm {T}^{\mathrm {t}}_{\text {XM}}$ and $\mathrm {T}^{\mathrm {t}}_{\text {XY}}$) which will become alternatively dominant at different divergence degrees.

We propose the following continuous time parameterization for Smith-Waterman:

$$\begin{aligned} \text{extend(t)} &= \log\mathrm{T}^{\mathrm{t}}_{\text{XX}},\\ \text{open(t)} &= \log\left(\mathrm{T}^{\mathrm{t}}_{\text{MX}}\right) + \log\left(1-\mathrm{T}^{\mathrm{t}}_{\text{XX}}\right) \\ &\quad-\log\left(\mathrm{T}^{\mathrm{t}}_{\text{XX}}\right),\\ \text{substitution score}\,\, S_{t}(\text{\textit{a,b}}) &= \log \frac{P_{t}(\text{\textit{a,b}})}{f_{a}\, f_{b}} = \log \frac{P_{t}(b\mid a) f_{a}}{f_{a}\, f_{b}} \\&= \log \frac{P_{t}(b\mid a)}{f_{b}} = \log \frac{\left(e^{t Q}\right)(\text{\textit{a,b}})}{f_{b}}. \end{aligned} $$

where the functions $\mathrm {T}^{\mathrm {t}}_{\text {MX}}$ and $\mathrm {T}^{\mathrm {t}}_{\text {XX}}$ are the time-dependent joint transition probabilities of the AFR model given in Fig. 4. The conditional probabilities are given by *P*_*t*_(*b*∣*a*)=(*e* ^*tQ*^)(*a,b*), where the *K*×*K* rate matrix *Q* is such that $\sum _{b=0}^{K-1} Q(\text {\textit {a,b}}) = 0$, for each residue 0<*a*<*K*, for an alphabet of size *K*, and has *f*_*b*_ as its saturation probabilities (that is, ${\lim }_{t\rightarrow \infty }\left (e^{t\,Q}\right)(\text {\textit {a,b}}) = f_{b}$).^2^

The standard BLAST parameterization (-11/-1/BLOSUM62) can be cast as a fixed-time instance of the previous parameterization as,

$$\begin{aligned} \text{extend}(t^{\star}) &\,=\,& -1 &= 2 \log_{2}\left(\mathrm{T}^{\star}_{\text{XX}}\right). \\ \text{open}(t^{\star}) &\,=\,& -11 &= 2 \log_{2}\frac{\mathrm{T}^{\star}_{\text{MX}}\left(1-\mathrm{T}^{\star}_{\text{XX}}\right)}{\mathrm{T}^{\star}_{\text{XX}}},\\ S_{t^{\star}}(\text{\textit{a,b}}) &=& 2\log_{2} \frac{P^{B62}(b\mid a)}{f_{b}} &= 2\log_{2} \frac{\left(e^{t^{\star} Q}\right)(\text{\textit{a,b}})}{f_{b}}, \end{aligned} $$

where *P* ^*B*62^ are the conditional probabilities corresponding to BLOSUM62, and $\mathrm {T}^{\star }_{\text {MX}}$ and $\mathrm {T}^{\star }_{\text {XX}}$ stand for the particular values of the corresponding time-dependent joint transition probabilities at a fixed and arbitrary time *t*=*t* ^⋆^.

We can rewrite the above equations as,

$$\begin{aligned} &\,\mathrm{T}^{\star}_{\text{XX}} = 2^{-1/2} &= 0.7017,\\ &\mathrm{T}^{\star}_{\text{MX}} = \frac{\mathrm{T}^{\star}_{\text{XX}}}{1-\mathrm{T}^{\star}_{\text{XX}}}\, 2^{-11/2} &= 0.0534,\\ &Q^{B62} = \log P^{B62}, \end{aligned} $$

where we assume with all generality that *t* ^⋆^=1, and the rate *Q* ^*B*62^ is the logarithm of the BLOSUM62 substitution matrix.

Under the AFR evolutionary model, we can rewrite (see Fig. 4),

$$\begin{aligned} &\,\mathrm{T}^{\star}_{\text{XX}} = \beta^{\textsc{lr}}_{\star} \,(1-r_{X}) + r_{X} &= 0.7017, \\ &\mathrm{T}^{\star}_{\text{MX}} = \beta^{\textsc{lr}}_{\star} \,(1-r_{M}) &= 0.0534. \end{aligned} $$

Not all parameters of the AFR model (*λ*,*μ*_*A*_,*r*_*X*_,*r*_*M*_) can be determined by the above conditions.

Introducing $\beta ^{\textsc {lr}}_{\infty } =\lambda /(\lambda +\mu _{A})$, which is the value of $\beta ^{\textsc {lr}}_{t} $ at infinite divergence, we have

$$\beta^{\textsc{lr}}_{t} = \beta^{\textsc{lr}}_{\infty} \frac{1-e^{-\mu_{A}\,t}}{1- \beta^{\textsc{lr}}_{\infty} \,e^{-\mu_{A}\,t}}. $$

Then, we can solve the above equations to obtain the values of the parameters of the AFR model given the fixed-time values $\mathrm {T}^{\star }_{\text {MX}}, \mathrm {T}^{\star }_{\text {XX}}$, and $\beta ^{\textsc {lr}}_{\infty } $ as,

(1) $$ \begin{aligned} &\mu_{A} = \log \frac{\beta^{\textsc{lr}}_{\infty} \,\left(1-\beta^{\textsc{lr}}_{\star} \right)}{(\beta^{\textsc{lr}}_{\infty} -\beta^{\textsc{lr}}_{\star})}, \\ &\,\ \ \lambda = \mu_{A}\,\frac{\beta^{\textsc{lr}}_{\infty} }{1-\beta^{\textsc{lr}}_{\infty} },\\ &r_{X} \,= \frac{\mathrm{T}^{\star}_{\text{XX}}-\beta^{\textsc{lr}}_{\star} }{1-\beta^{\textsc{lr}}_{\star} }, \end{aligned}   $$

where

$$\beta^{\textsc{lr}}_{\star} = \frac{\mathrm{T}^{\star}_{\text{MX}}}{1-r_{M}}, $$

and $\beta ^{\textsc {lr}}_{\infty } $ and *r*_*M*_ are still undetermined.

In order to infer positive valued rates such that *λ*≤*μ*_*A*_ (so that *p* ^LR^=*λ*/*μ*_*A*_<1), we need to impose the conditions $\beta ^{\textsc {lr}}_{\star } < \beta ^{\textsc {lr}}_{\infty } < 0.5$. The Bernoulli parameter *r*_*X*_ is properly parameterized as long as $\mathrm {T}^{\star }_{\text {XX}}>\beta ^{\textsc {lr}}_{\star } $, which is usually the case, and in particular it is satisfied by this parameterization. The two remaining parameters *r*_*M*_ and $\beta ^{\textsc {lr}}_{\infty } $ are not constrained by the fixed-time point values of -11/-1/BLOSUM62. Those two parameters can be estimated from data.

For a given set of values of the four parameters of the AFR model (*μ*_*A*_,*λ*,*r*_*X*_,*r*_*M*_), we can calculate the open and extend costs at any other time given the expressions for the AFR model (Fig. 4). The continuous-time affine-gap and substitution score functions are (in half bits)

(2) $$ \begin{aligned} &\text{extend}(t) = 2\,\log_{2}\mathrm{T}^{\mathrm{t}}_{\text{XX}}\\ &\qquad\qquad= 2\,\log_{2}\left(\beta^{\textsc{lr}}_{t} (1-r_{X}) + r_{X}\right), \\ &\ \ \ \text{open}(t) = 2\, \log_{2}\frac{\mathrm{T}^{\mathrm{t}}_{\text{MX}}(1-\mathrm{T}^{\mathrm{t}}_{\text{XX}})}{\mathrm{T}^{\mathrm{t}}_{\text{XX}}} &\\ &\qquad\qquad= 2\, \log_{2}\frac{\beta^{\textsc{lr}}_{t} \left(1-\beta^{\textsc{lr}}_{t} \right)(1-r_{X})(1-r_{M})}{\beta^{\textsc{lr}}_{t} (1-r_{X}) + r_{X}},\\ &\ \ \ \,S_{t}(\text{\textit{a,b}}) = 2\,\log_{2} \frac{P_{t}(\text{\textit{a,b}})}{f_{a} f_{b}} &\\ &\qquad\qquad= 2\,\log_{2} \frac{\left(e^{t\,Q^{B62}}\right)(\text{\textit{a,b}})}{f_{b}}. \end{aligned}  $$

Homology programs such as SSEARCH and FASTA provide a handful of different substitution matrices at different percentage identity with their corresponding recommended open and extend penalties, which have been estimated by numerical testing [28, 36]. Here for demonstration, we fix the values of the remaining free parameters *r*_*M*_ and $\beta ^{\textsc {lr}}_{\infty } $ such that they produce a relatively good agreement with the empirical values collected for SSEARCH and FASTA in [28]. We use the values *r*_*M*_=0.75, and $\beta ^{\textsc {lr}}_{\infty } =0.30$, which result in $\beta ^{\textsc {lr}}_{\star } = 0.2134$. Applying these specific values into the algebraic expression of the AFR model parameters in Eq. 1, we obtain,

$$\begin{array}{@{}rcl@{}} \mu_{A} &=& 1.0023,\\ \lambda &=& 0.4296,\\ r_{X} &=& 0.6276,\\ r_{M} &=& 0.7500. \end{array} $$

**Fig. 6:**
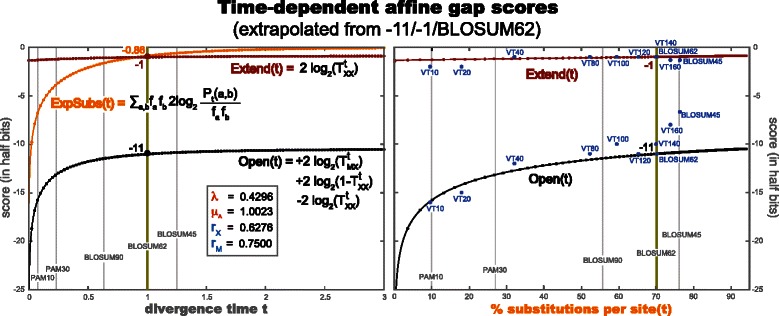
Explicit continuous-time affine-gap cost functions (based on the AFR evolutionary model) extrapolated from the commonly used -11/-1 gap cost of standard sequence/sequence Smith-Waterman comparisons. Time-dependent synchronized parameterization of the substitution score with its corresponding gap open and gap extend costs based on the AFR evolutionary model. The parameterization is such that for time *t*=1, it corresponds to a gap open cost of -11, a gap extend of -1, and the BLOSUM62 scoring matrix. In the left figure, the gap-open and gap-extend functions and the expected substitution score $\left (\sum _{\textit {ab}} f_{a} f_{b} \log \frac {P_{t}(\text {\textit {a,b}})}{f_{a} f_{b}}\right)$ are depicted as a function of the divergence time. In the right figure, the gap-open and gap-extend functions are depicted as a function of the fraction of substitutions at that time, defined as $\sum _{a\neq b}P_{t}(\text {\textit {a,b}})$. The blue dots correspond to gap-open and gap-extend values empirically determined for SSEARCH with different substitution matrices in [28]. (The gap scores for the VT160 and BLOSUM50 matrices originally given in 1/3 bits have been rescaled to half bits)

The continuous-time affine-gap costs for this particular parameterization, and their correspondence to the empirical discrete values used by FASTA as described in [28] are given in Fig. 6. A collection of particular point values for the “open” and “extend” functions for notable evolutionary distances is given in Table 2.

**Table 2:** Several point values for the affine gap cost continuous-time functions open(t) and extend(t) for time instances associated to well known substitution matrices. The analytic functions are given in Eq. 2 and depicted in Fig. 6. The parameter values are: $\mu _{A}=1.0023,\lambda =0.4296,r_{X}=0.6276,r_{M}=0.7500,\beta ^{\textsc {lr}}_{\infty } =0.3000,\beta ^{\textsc {lr}}_{\star } =0.2134$. The divergence parameter t has been normalized to BLOSUM62

	% Substitutions	Divergence	Open	Extend
	per site	time t		
PAM10/VT10	10.0	0.074	–15.79	–1.29
VT20	17.9	0.141	–14.21	–1.25
PAM30	27.1	0.229	–13.14	–1.21
VT40	32.0	0.283	–12.73	–1.18
PAM70	49.8	0.520	–11.72	–1.10
VT80	52.2	0.567	–11.60	–1.09
BLOSUM90	55.8	0.633	–11.46	–1.07
VT100	59.4	0.709	–11.33	–1.05
BLOSUM80	60.9	0.741	–11.28	–1.05
VT120	65.2	0.851	–11.14	–1.02
VT140	70.0	0.995	–11.00	–1.00
BLOSUM62	70.1	1.000	–11.00	–1.00
BLOSUM50	72.9	1.099	–10.93	–0.99
VT160	73.8	1.135	–10.90	–0.98
PAM120	74.9	1.180	–10.88	–0.98
BLOSUM45	76.3	1.245	–10.84	–0.97

By construction and for this particular set of parameters of the AFR model, the continuous-time functions valued at *t*=1 reproduce the values of the the standard -11/-1/BLOSUM62 parameterization of BLAST and SSEARCH.^3^

### Alignment accuracy using explicit evolutionary models

Profile methods for sequence homology search and alignment, unlike standard sequence/sequence comparison methods, do not assume any equivalence between deletions and insertions. Consequently, the evolutionary models compatible with profile HMMs such as the AIF and AGA model are not reversible and cannot be incorporated into the symmetric pair HMM of Fig. 4. We are interested in investigating the potential of nonreversible evolutionary models but that are still compatible with standard three-state pair HMMs, such as AIF and AGA.

We have implemented an alignment algorithm (named e2msa) that uses an explicit evolutionary model and standard pair HMMs. Having a model with a continuous variable parameterization allows us to compare the performance of the model at different parameterizations describing different branch lengths, that is, different degrees of sequence similarity. The e2msa alignment algorithm also allows us to compare the performance of the different evolutionary models. All evolutionary models described in Table 1 have been implemented as part of e2msa. We give a detailed description of the program e2msa and the training of the evolutionary parameters in the ‘Methods’ section.

Next we test the performance (for alignment accuracy and homology coverage in pairwise alignments) of a short-branch parameterization and a long-branch parameterization, compared to an optimal-branch parameterization in which the divergence time is the one that maximizes the score of the sequences being compared. We expected to see that optimal-branch parameterized models produce more accurate alignments.

#### A fixed *long-branch* parameterization is sufficient to align *global* homologies

We created an alignment benchmark composed of a mixture of structural pairwise alignments (the reference alignments from databases SABmark and PREFAB), as well as conserved (≥ 40 % identity) pairwise alignments selected at random from random Pfam families. All sequences in this benchmark are full-length homologies. Details about this large benchmark (more than 35,000 pairwise alignments) named the “Global Homology set” are provided in the ‘Methods’ section. While the alignments in SABmark and PREFAB are very divergent (all are less than 50 % identity), due to the Pfam contribution, the “Global Homology set” covers all ranges of percentage identities, with at least 100 alignments in each of the more divergent identity ranges 80–85 %, 85–90 %, 90–95 % and 95–100 %, and many more in the other less divergent ranges.

**Fig. 7:**
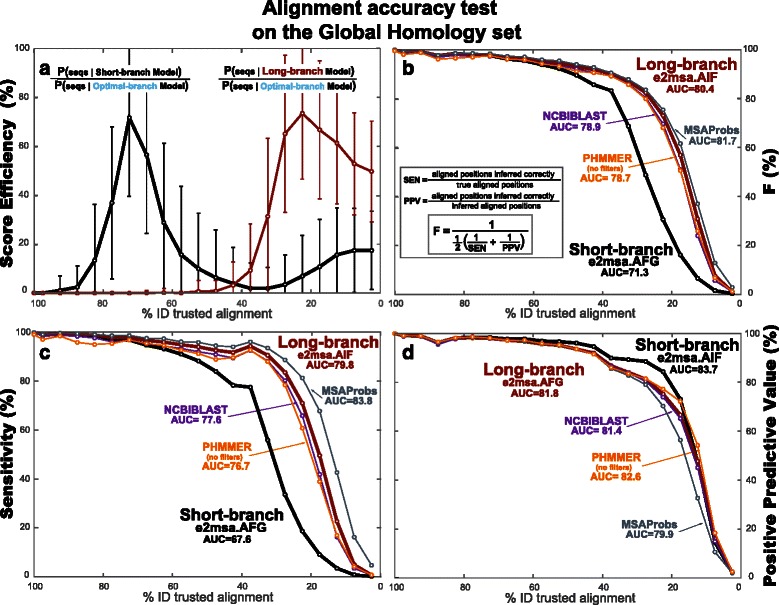
The score efficiencies for a long-branch versus a short-branch parameterized model have no correspondence with alignment accuracy for the Global Homology set. **a** For the *long-branch* and *short-branch* parameterizations of the AIF model in e2msa, we present their score efficiency as a function of the percentage identity of the alignments. Alignments are binned in 5 % identity groups (relative to the trusted alignments). For each identity bin, the mean and standard deviation of the score efficiency are calculated. **b** For the *long-branch* and *short-branch* models, we present the accuracy of the alignments inferred by e2msa. Alignment accuracy is calculated using the F measure that combines sensitivity (the fraction of aligned positions inferred correctly) and positive predictive value (the fraction of inferred aligned positions that are correct). We present comparisons with the methods NCBIBLAST, PHMMER, and MSAProbs. Panels (**c**) and (**d**) report the sensitivity (SEN) and positive predictive value (PPV) measures respectively for completeness

In Fig. 7, we compare two single fixed-branch parameterizations: a “long-branch” parameterization that (by sampling from the model) produces alignments with an average percentage identity of 27 % (similar to that of BLOSUM62), and a “short-branch” parameterization with 71 % average identity alignments (similar to PAM30). We calculate alignment accuracy by measuring sensitivity (fraction of aligned positions that are inferred correctly), and positive predictive value (PPV or fraction of predicted aligned positions that are correct). The two measures can be combined in one single measure, the F value, the harmonic mean of sensitivity and positive predictive value. As a single measure that conveys information about a given method for alignments at all percentages of identity, we also provide the area under the curve (AUC) for a given measure (sensitivity, positive predictive value, or F).

Alignments are binned by percentage identity (here we use 5 % identity bins). Alignment accuracy measures are calculated for each identity bin independently of of any of the other bins.

For the two fixed-branch models, we also calculate (for each percentage identity bin) the mean score efficiency (based in a similar measure introduced for time-dependent substitution matrices [4]) which corresponds to the fraction of the maximal information of the evolutionary model that is captured by a single fixed-branch parameterization. The score efficiency is the ratio of the probability of the sequences given the e2msa model (the Forward score) for the fixed-branch parameterization divided by the Forward score of the optimal parameterization.

We observe in Fig. 7, by comparing the score efficiency of the long-branch and short-branch parameterizations, that the two models are tuned to the alignment’s divergence: the long-branch parameterization produces more efficient scores for more divergent alignments, while the short-branch parameterization produces better scores for less divergent alignments. However, contrary to our expectations, this selective score efficiency does not translate into alignment accuracy: the *long-branch* parameterization which (as expected) performs better for alignments of divergent sequences, it also performs well (comparable to a short-branch parameterization) for alignments of very conserved sequences. This result can be seen in Fig. 7 b for the composite F measure. We have also included the two components of F (sensitivity and positive predictive value) in Fig. 7 c and d respectively for completeness. We have also tested homology coverage, which produces comparable results to those of alignment accuracy.

We have performed the same experiments using several different evolutionary models in e2msa. Results are presented in Table 3 for alignment accuracy (and in Table 4 for homology coverage). Evolutionary models with more parameters tend to perform better (unsurprisingly). Fragment models perform better than their corresponding single-residue counterparts. The two models compatible with profile HMMs (the AIF and the AGA models) perform similarly to each other, and to the AFG model. The AFR model performs comparably to TKF92, and it has the advantage of allowing a symmetric pair HMM implementation.

**Table 3:** Alignment accuracy for global and local homologies of different evolutionary models implemented under the e2msa local alignment algorithm

	Alignment Accuracy					
	[ AUC for F measure (%)]					
Method	Global homology set			Local homology set		
	parameterization			parameterization		
	short	long	optimal	short	long	optimal
e2msa.afg	71.4	**80.4**	80.3	68.2	68.2	**73.6**
e2msa.aga	71.4	**80.4**	80.1	68.2	67.3	**73.6**
e2msa.aif	71.3	**80.4**	80.2	68.1	68.3	**73.3**
e2msa.tkf92	71.2	**80.0**	79.9	68.1	68.2	**73.4**
e2msa.afr	71.7	**80.0**	79.8	68.1	68.2	**73.3**
e2msa.aali	71.0	**78.7**	78.6	67.9	66.4	**72.7**
e2msa.tkf91	69.5	**75.4**	74.5	66.2	69.1	**70.7**
PHMMER (no filters) SSEARCH		78.7			72.9	
(BLOSUM62, -11/-1)		80.0			71.7	
NCBIBLAST		78.9			68.4	
MSAProbs		81.7			NA	
MUSCLE		80.8			NA	

**Fig. 8:**
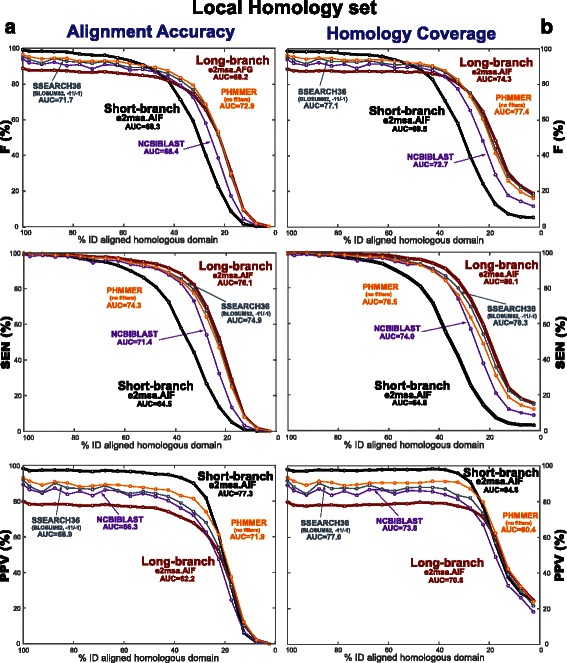
Alignment accuracy and homology coverage for the Local Homology set of subregions embedded in nonhomologous sequence. Panel (**a**) displays the measures of alignment accuracy. Panel (**b**) displays coverage measures. The long-branch and short-branch parameterizations are identical to those described in Fig. 7. The results presented correspond to the evolutionary model AIF using the alignment program e2msa

The “Global Homology set” is the one used in Fig. 7. The “Local Homology set” is the one used in Fig. 8. The e2msa algorithm was run in local mode, and with three different parameterizations: two at a fixed branch length (a short-branch and a long-branch parameterization, introduced in Fig. 7), and a variable optimal-time parameterization that uses for each homology the branch length that optimizes the probability of the sequences given the model. The rate parameters for all evolutionary model were obtained using the same training set “Pfam.seed.S1000.sto”. For all experiments, alignments are binned in 5 % identity groups, and the total F measure for one bin is calculated adding all alignments in that bin. In order to provide one single number, we report the area under the curve (AUC) for the F measure of alignments covering all identity ranges. For comparison, we provide results for other standard methods. Methods have been ranked by their combined performance in both sets. Methods such as MSAProbs and MUSCLE work only in “global” alignment mode, and they are not appropriate to detect local homologies

In bold, we indicate the best performing of the three alternative parameterizations

**Table 4:** Homology coverage performance for global and local homologies of different evolutionary models implemented under the e2msa local alignment algorithm

	Homology Coverage					
	[AUC for F measure (%)]					
Method	Global homology set			Local homology set		
	parameterization			parameterization		
	short	long	optimal	short	long	optimal
e2msa.afg	74.3	**86.9**	86.7	69.5	73.9	**78.2**
e2msa.aga	74.4	**87.3**	86.5	69.6	73.1	**78.0**
e2msa.aif	74.0	**86.8**	86.6	69.5	74.3	**78.2**
e2msa.tkf92	74.6	**86.5**	86.0	69.4	73.8	**77.7**
e2msa.afr	74.6	**86.4**	86.0	69.3	73.6	**77.5**
e2msa.aali	73.9	**86.7**	85.4	69.6	73.3	**77.6**
e2msa.tkf91	72.8	**79.6**	78.0	67.7	72.0	**72.6**
SSEARCH (BLOSUM62, -11/-1)		86.7			77.1	
PHMMER (no filters)		83.3			76.7	
NCBIBLAST		85.0			72.7	
MSAProbs		99.3			NA	
MUSCLE		99.3			NA	

In bold, we indicate the best performing of the three alternative parameterizations

From these results, one would be tempted to conclude that the best strategy is to always use a long-branch parameterization and never bother with implementing an explicit evolutionary model which carries the additional expense of optimizing the branch length. However, there is a common scenario not addressed by this benchmark of global homologies that we investigate next, the reported artifact of *overextension* of local alignments into flanking nonhomologous sequence when using a long-branch parameterized method [19, 27, 28].

#### A fixed *short-branch* parameterization reduces nonhomologous overextension

From the “Global Homology set”, we have constructed a “Local Homology set” with the same number of alignments, but where we have preserved only a small part of the original global alignment (50 positions on average), and have replaced the rest of the original homologous sequences with random sequence (details are given in the ‘Methods’ section).

We perform the same alignment accuracy benchmark for this “Local Homology set”. Results are presented in Fig. 8 a. Unlike the situation for the “Global Homology set” described in Fig. 7, we observe that in the presence of local homologies the short-branch parameterization performs better (as given by the F measure) than the long-branch parameterization for more conserved homologies, while the long-branch parameterization performs better for more divergent homologies.

What makes the local homology case different is that the inferred alignment can extend into the nonhomologous flanking regions. The alignment accuracy PPV measure penalizes both homologous residues that are improperly align to other homologous residues as well as aligned positions that include nonhomologous residues. In order to disentangle these two effects, we calculated measures of homology coverage. Coverage measures isolate the nonhomologous overextension problem because they penalize nonhomologous positions that are included in the alignment, but they do not penalized homologous positions misaligned to other homologous positions. Coverage sensitivity calculates the fraction of homologous positions that are included in the alignment, and coverage positive predictive value measures the fraction of aligned positions that are actually homologous. The coverage F measure is the harmonic mean of its SEN and PPV.

Homology coverage results are presented in Fig. 8 b. For close relationships (40 % identity or more), we observe that coverage measures are similar to those of alignment accuracy. Thus, the low alignment accuracy PPV observed for the long-branch parameterization (relative to that of the short-branch parameterization) is mostly associated with alignment overextension. For distant relationships (less than 40 % identity), the poorer values for alignment accuracy relative to those of coverage are mostly due to misalignment of homologous residues.

Both for alignment accuracy and homology coverage, the sensitivity of the short-branch parameterization is uniformly below that of the long-branch parameterization. However the consistently better PPV for the short-branch parameterization more than compensates for the loss in sensitivity. The result is that for the F measure (the harmonic mean of sensitivity and PPV) there is a crossover such that the short-branch parameterization is better for close relationships and the long-branch parameterization is better for distant relationships.

Regarding the behavior of the different affine evolutionary models for the Local Homology set, we observe the same trends as as for the Global Homology set. In particular, the AIF and AGA models compatible with profile HMMs perform similarly to each other and to the AFG model, and the AFR model performs similarly to the TKF92 model. See Table 3 for alignment accuracy of the different evolutionary models, and Table 4 for homology coverage.

#### A variable *optimal-branch* parameterization is best to align local homologies

An explicit evolutionary model allows us to optimize for the parameterization that best suits a given homology comparison. The optimal-branch parameterization of e2msa uses a simple line-optimizer to select the branch length that achieves the best probability for the compared sequences summing to all possible alignments.

**Fig. 9:**
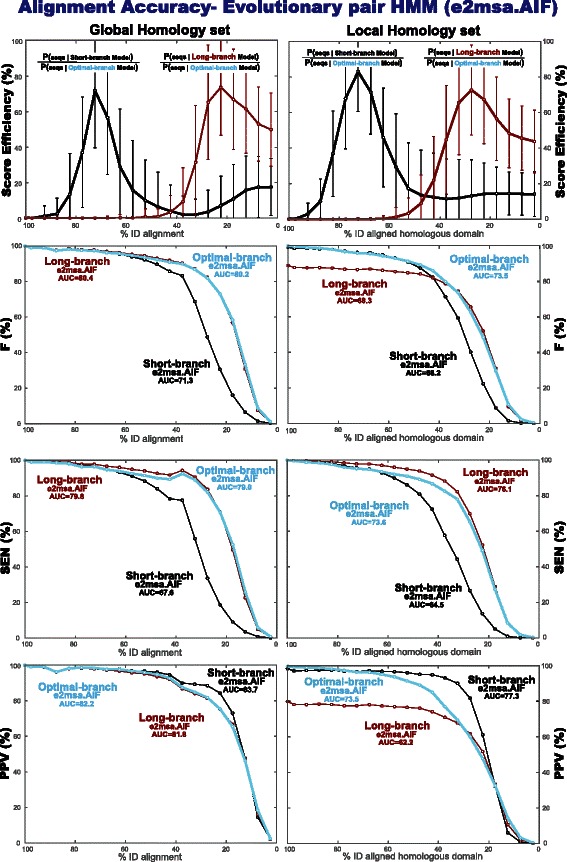
Alignment accuracy of the optimal-branch e2msa.AIF method both for the Global and Local homology sets. The results for the short-branch and long-branch parameterizations for the Global (Local) homology set are the same as those provided in Fig. 7 (Fig. 8 a) now placed in reference to the optimal-branch parameterization

**Fig. 10:**
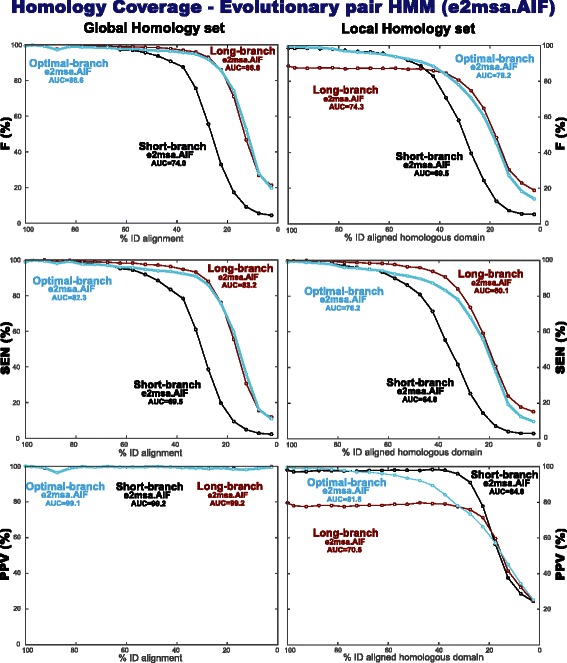
Homology coverage benchmark: performance of the optimal-branch e2msa.AIF method both for global and local homologies. The reason why the PPV for the Global Homology set is not 100 % for in all case, is because the structural alignments from the benchmark PREFAB include some positions in lowercase which are not supposed to be considered aligned, thus there is the option of some small overextension into those positions. The results for the short-branch and long-branch parameterizations for the Local homology set are the same as those provided in Fig. 8 b now placed in reference to the optimal-branch parameterization

We have applied the optimal-branch parameterization of e2msa to both the Global and Local Homology sets. Results for alignment accuracy are presented in Fig. 9. As expected, for the Global Homology set, the optimal-branch model performs almost identically to the long-branch model for all degrees of sequence divergence. For the Local Homology set, on the other hand, the optimal-branch model follows the short-branch model for more conserved sequences, and follows the long-branch model for more divergent sequences, thus combining the best of both regimes. Results for homology coverage (Fig. 10) are similar to those of alignment accuracy.

## Conclusions

We have identified and implemented probabilistic models of biological sequence evolution that provide an affine treatment of insertions and deletions, and that can be compactly described as a standard three-state pair HMM in which the emission and transition probabilities are continuous time functions depending on a small number of independent rate parameters. An unexpected difficulty is the realization that macroscopic inserts with geometrically distributed lengths force quite constrained and unrealistic microscopic events such as indivisible fragments; and that for instance, single event geometrically distributed insertions do not produce geometrically distributed inserts. One of the evolutionary models (the AFR model) is compatible with the symmetric pair HMMs that treat insertions and deletions indistinguishably used routinely in sequence/sequence comparisons. Two of the evolutionary models (AIF and AGA) are compatible with standard profile HMMs for which there is a set of independent rate parameters for each position in the profile.

A continuous time evolutionary model, including a model of indels, allows us to optimize the parameterization of a profile/sequence or sequence/sequence comparison to match the apparent divergence time. Using a single alignment program implementing pair-HMMs (e2msa), we asked what gain (if any) this could bring. Our results show that the most important benefit of using an optimal-branch parameterization is the reduction of homologous overextension artifacts in which non homologous regions become part of the alignment and are treated as homologous. For global homologies with no risk of overextension, a fixed long-branch parameterization is the most economical choice to provide the best possible performance. Optimal-branch parameterized models could improve iterative methods such as JACKHMMER or PSI-BLAST in which it is important to avoid overextension of hits in the early stages of the search. Another advantage of a short-branch parameterization that we can anticipate, but have not tested in this manuscript, is the detection of very short homologies such as in the analysis of short metagenomic reads, although preliminary results suggest this effect would only be relevant for ORFs shorter than 10 aa.

Because e2msa implements several different evolutionary models, we can conduct a level comparison of those models keeping the rest of the details of the alignment algorithm constant. We observe that the AIF and AGA evolutionary models compatible with profile HMMs are amongst the best performing model in a pairwise alignment accuracy benchmark of both global and local homologies.

The AIF and AGA models are not applicable to some specific profile HMMs such as HMMER that disallow transitions between Delete and Insert states (named Plan7, for using only 7 transitions for a given profile position). HMMER’s original design was purposely non-evolutionary, favoring structural alignments where nonhomologous positions may appear the same alignment column as they occupy the same nonconserved region in between structurally well defined conserved regions. Constructing an evolutionary version of HMMER is going to require changing the architecture of the HMM to include all possible transitions from one profile position to the next (a Plan9 profile HMM).

In an evolutionary profile HMM, since we want to obtain position-specific scores, we require position-specific rates both for the substitution and insertion/deletion process. We anticipate replacing the standard profile training method by using a mixture of HMM rates trained in a large protein domain database that would replace the use of mixture Dirichlet priors, a phylogenetic tree that would replace sequence weighting, and the evolutionary time that would replace entropy weighting. It remains an open question whether it is worth implementing an evolutionary profile HMM to improve training (by removing the use of Dirichlet priors, sequence and entropy weighting), when the method is going to be used mainly with a very long branch parameterization.

## Methods

### e2msa: a pair HMM alignment algorithm implementing explicit evolutionary models

In order to compare the different evolutionary models while maintaining other variables equal, and to measure the effect of using an explicit evolutionary model on alignment accuracy, we have implemented a pairwise or multiple sequence alignment program (named e2msa) that can adopt any of the evolutionary models described in Table 1.

Constructing evolutionary alignments using any of the models in Table 1 simply requires that in the Forward and Viterbi algorithms of a standard local pair HMM described in [3], one replaces the constant emission and transition probabilities with continuous time functions dictated by the evolutionary model. A pair HMM describes the probability of an ancestral sequence and one of its descendants. Thus, a pair HMM can only be used to align two extant sequences for reversible evolutionary model such as the AFR model (where one sequence can be taken as the ancestor of the other one with all generality). A profile HMM on the other hand describes the relationship between an extant sequence and the (ancestral) profile. Thus, profile HMMs do not care about reversibility, and in fact the evolutionary models (AIF and AGA) compatible with profile HMMs are nonreversible. In order to test non reversible evolutionary models using pair HMMs, we need to calculate the probability of two extant sequences being generated by a unknown common ancestor, each of them according to a standard pair HMM.

**Fig. 11:**
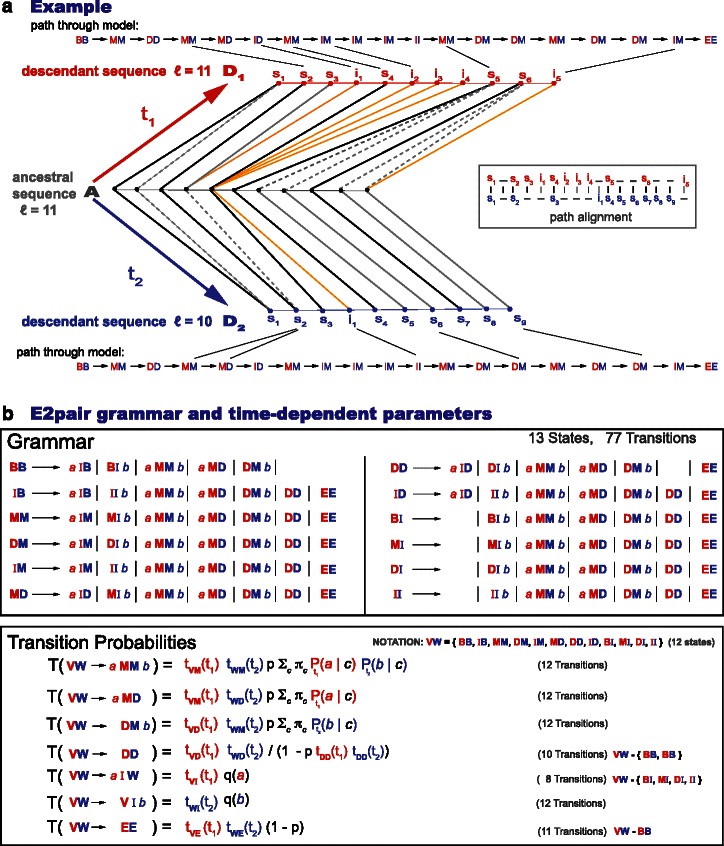
Model for aligning two extant sequences related by a common ancestor using an affine gap-cost and generally not reversible evolutionary model. **a** In the example, two extant sequences (marked with red and blue) are aligned according to an evolutionary history that involves an ancestral sequence of 11 residues, 5 aligned positions, one double deletion, and 11 gaps. Some of the gaps correspond to ancestral residues deleted in either of the sequences, and some of the gaps correspond to insertions (relative to the ancestor) in one of the two extant sequences. Many different choices of ancestral sequence and evolutionary histories (in addition to this example) contribute to that particular alignment. The E2pair model describes the probability associated to each of those processes. **b** The E2pair model grammar is described here. A particular history can be derived in only one way by the grammar (an unambiguous grammar). Since the ancestral sequence is an unknown, the model sums over all possible ones. The transition probabilities t_VW_(*t*) are given by one of the evolutionary models specified in Table 1 evolutionary model (see also Fig. 2 and Fig. 3). The emission probabilities include a time-dependent substitution matrix *P*_*t*_(*a*∣*b*), a residue distribution for emitting inserted residues *q*_*I*_(*a*), and another one for ancestral residues *π*(*a*). There is also the geometric parameter *p* describing the distribution of ancestral sequence lengths. Since double deletions are not observed, the algorithm also sums over all of them. The factor 1/(1−*p*t_DD_t_DD_) that appears in all the transitions into the DD state, corresponds to summing over all possible DD→DD…→DD transitions, given by $\sum ^{\infty }_{n=1} (p \mathrm {t}_{\text {DD}}\mathrm {t}_{\text {DD}})^{n}$

**Fig. 12:**
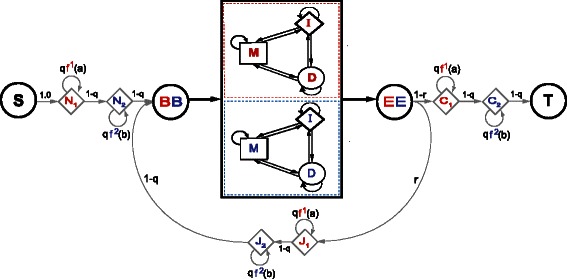
The local E2pair model. In the local version of the E2pair model, unaligned regions can occur at the N or C-terminus or in between two homologous regions. The length of the unaligned regions is controlled by the Bernoulli parameter *q* (the expected length of a nonhomologous region is *q*/(1−*q*)), and the expected number of nonhomologous regions is *r*/(1−*r*). The residue frequencies *f* ^1^ and *f* ^2^ are background distribution for the nonhomologous regions. In between the BB and the EE states a homologous region between the two sequences occurs according to the E2pair global model in Fig. 11 b. The global alignment case corresponds to setting *q*=*r*=0

We have implemented the so-called E2pair algorithm to calculate the probability of two sequences being related by a common ancestor, as described in Fig. 11. The algorithm sums over all possible evolutionary histories and ancestral sequences. In Fig. 11 a, we provide an example of two extant sequences related by one particular ancestral sequence and one particular evolutionary history. In Fig. 11 b, we provide a full description of the E2pair model and its time-dependent parameters, where the time dependencies are given by a specific evolutionary model. The E2pair dynamic programming algorithm calculates *P*(*s*_1_,*s*_2_∣evomodel,*t*_1_,*t*_2_), that is, the probability of two sequences *s*_1_,*s*_2_ both descend from the same (unknown) common ancestor after times *t*_1_ and *t*_2_ respectively. The E2pair model does not assume that all the extent of the sequences being compared have to be homologous. By default as described in Fig. 12, the E2pair model allows for nonhomologous regions flanking the (possibly more than one) homologous regions. We refer to this as the local mode of the E2pair model. Under a particular parameterization described in Fig. 12, the E2pair model can force the two sequences to align in full; we refer to that as the global mode parameterization.

**Fig. 13:**
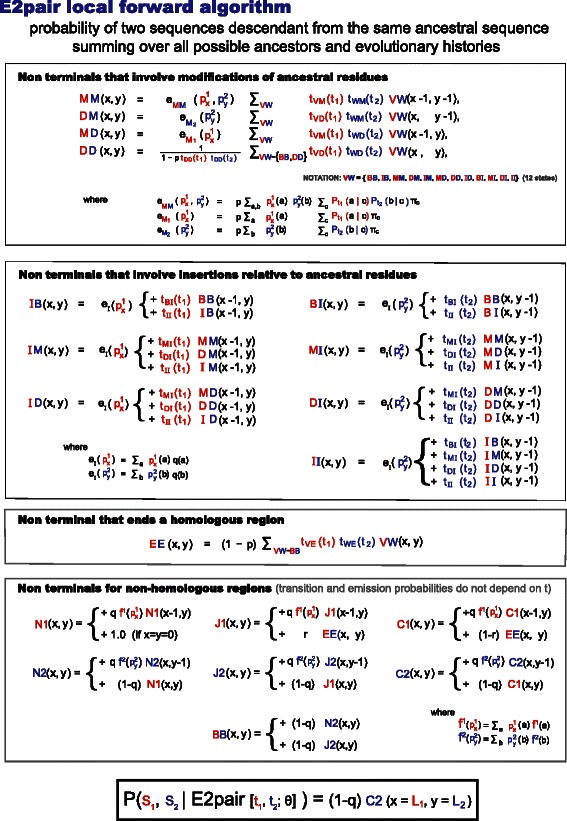
Dynamic programming algorithm for the E2pair model. Dynamic programming algorithm to calculate the probability of two sequences related by a common ancestor by summing to all ancestors and to all evolutionary histories. The algorithm behaves as *O*(*l*1×*l*2) both in time and memory for two sequences of lengths *l*1 and *l*2. The parameters of the model have been described in Fig. 11. The algorithm assumes that the internal nodes are “profile” sequences, that is, sequences that instead of a fixed residue per position, they have a probability distribution of residues for each position ${p^{1}_{x}}(a),{p^{2}_{x}}(a)$. This generalization becomes useful when we extend this pairwise algorithm to perform a progressive alignment, and we align internal nodes of the tree. Internal node sequences are estimated from the E2pair algorithm by the optimal posterior path through the model

The Forward algorithm for the E2pair model is described in detail in Fig. 13. It has a complexity of *O*(*l*1×*l*2) both in time and memory for two sequences of lengths *l*1 and *l*2.

The e2msa method can be run at a fixed evolutionary distance (fixed branch) or using variable branch lengths obtained by optimizing the probability of the sequences being compared given the model. The e2msa method can be run in local or global mode. All experiments in this manuscript are run in local mode.

#### Training of rate parameters

The rate parameters of the evolutionary model are an input to the e2msa program. In all experiments performed here, e2msa uses evolutionary rate parameters derived from a training set of 1000 trusted pairwise alignments obtained at random from Pfam. This training set is independent of any other Pfam family used in the alignment benchmarks. The training set file is named “Pfam.seed.S1000.sto”, and it is included as part of the Additional file 2.

The rate parameters are obtained by optimizing the total probability of the sequences in the training set given the evolutionary model using a gradient descent optimization method implemented in the program e2train. For the emission probabilities, we constructed a rate matrix (scaled to one substitution per site) derived from the BLOSUM62 substitution matrix. In the Additional file 2, we provide evolutionary parameters (both the rates and the Bernoulli geometric parameters) trained on the same training set (“Pfam.seed.S1000.sto”) for all evolutionary models given in Table 1.

### Alignment accuracy benchmark

The alignment benchmark used in Fig. 7 consists of a total of 36,484 global homology pairwise alignments including the whole reference sets of SABmark (29,756 alignments) [38] and PREFAB (1682 alignments) [39], as well as a collection of 5043 pairwise alignment selected each from a random Pfam seed alignment, and such that these selected families are different from those used to generate the Pfam training set. Because standard alignment benchmarks like SABmark and PREFAB tend to select for more divergent alignments, we used Pfam to include more closely related alignments. For the Pfam alignments, we required at least 40 % identity between the sequences. The result is a large collection of trusted pairwise alignments of global homologies representing all degrees of sequence diversity. We refer to this set as the **“Global Homology set”**, and it is available in the Additional file 2.

In the “Global Homology set”, all 5 % percentage identity bins in which we report results include at least 100 alignments. The length of the sequences in the “Global Homology set” is quite similar in all identity bins, with mean and standard deviation that range from 199 +119 nts for the alignments in the 0–5 % identity bin, to 187 +/- 125 nts for the alignments in the 90–100 % identity bin. The absolute range of sequence lengths is 7 nts to 2196 nts.

Derived from the “Global Homology set”, we construct a set of local homologies. For each global homology alignment, we preserve just a section of the original alignment. The homologous fragments have lengths normally distributed around 50 positions, and are selected from a random starting position in the alignment. The original homologous residues not part of the selected homology fragment are replaced with an identical number of nonhomologous residues taken at random from UniProt and randomized by single-residue shuffling. We further require that the nonhomologous regions have to be at least a fifth of the total alignment length. The result is a collection of local homology alignments that relative to the original “Global Homology set” has a similar number of alignments (36,404) with sequences of identical length, but where we only preserve 20 % of the total number of homologous positions present in the original set. We refer to this set of alignments as the **“Local Homology set”**. The observed average length of the homologous regions in this set is 49 ± 10 amino acids. The “Local Homology set” is also available as part of the Additional file 2.

#### Measure of alignment identity

For two aligned sequences, their percentage identity is calculated as the ratio of identical positions divided by the minimum length of the two sequences. We calculate multiple alignment identity as the average percentage identity of the alignment’s pairwise comparisons.

#### Measures of alignment accuracy

We report alignment accuracy by pairwise comparison. For each pair of aligned sequences, we measure alignment accuracy by its sensitivity (SEN_A_) and positive predictive value (PPV_A_)

$$\begin{aligned} \text{SEN}_{\mathrm{A}}=\frac{\text{Aligned Positions Inferred Correctly}}{\text{True Aligned Positions}}, \quad\quad \text{PPV}_{\mathrm{A}}=\frac{\text{Aligned Positions Inferred Correctly}}{\text{Inferred Aligned Positions}}. \end{aligned} $$

Sometimes for simplicity, we use the F measure (the harmonic mean of the SEN and PPV [40]) as a proxy for prediction accuracy,

(3) $$ \mathrm{F}_{\mathrm{A}}=\frac{1}{\frac{1}{2}\,\left(\frac{1}{\text{SEN}_{\mathrm{A}}} + \frac{1}{\text{PPV}_{\mathrm{A}}}\right)}.   $$

In this work, the alignment sensitivity (SEN_A_) also referred to as the “SP-Score”, and positive predictive value (PPV_A_) also referred to as the “Modeler Score” have been calculated using the program FastSP v1.6 [41].

#### Measures of homology coverage

We measure homology coverage performance by its sensitivity (SEN_C_) and positive predictive value (PPV_C_). For a predicted multiple alignment of some embedded homologous regions, we define,

$$\begin{aligned} \text{SEN}_{\mathrm{C}}&=\frac{\text{\# of homologous residues aligned}}{\text{\# of homologous residues}},\\ \quad\quad \text{PPV}_{\mathrm{C}}&=\frac{\text{\# of homologous residues aligned}}{\text{\# of aligned residues}}. \end{aligned} $$

Similarly to alignment accuracy, the F measure of coverage (F_C_) is introduced as in Eq. (3).

Differently from alignment accuracy, the coverage measures do not penalize homologous positions aligned incorrectly to other homologous positions.

### Software implementations and availability

We provide the ANSI C source code for the programs e2msa (to produce a pairwise or progressive multiple sequence alignment using the E2pair algorithm), e2train (for training the evolutionary parameters of the pair-HMM), e2sim (for generating aligned sequences according to an evolutionary model and an arbitrary phylogenetic tree), and e1sim (to evolve sequences according to the different evolutionary models). All programs accept under one single implementation all the evolutionary models described in Table 1.

The ANSI C source code for programs e2msa, e2train, e2sim, and e1sim is freely available available under the GNU General Public License (GPL) from eddylab.org. All programs use the packages HMMER [21] and EASEL (S.R. Eddy, unpublished) as libraries, which are also provided under the same license.

The source code for the above programs, as well as the Perl scripts used to generate the experiments in this work and the results of the experiments have been collected in a tarball available as part of the Additional file 2.

#### Software and database versions used

We used the following versions of programs: NCBIBLAST 2.2.26+, [42], SSEARCH 36.3.6d [20], MSAProbs 0.9.7 [43], MUSCLE 3.8.31 [44], and FastSP v1.6 [41].

The VT amino acid substitution score matrices [45] used in Fig. 6 were created using the Perl script “vt_scores.pl” written by K. Kneutgen and T. Mueller (May 2002), and provided to us by courtesy of W.R. Pearson. The VT conditional probability matrices have been generated using a modified version of the original script, named “vt_scores_modER.pl” which also provides the target amino acid frequencies, and the percentage identity. The scripts “vt_scores.pl”, “vt_scores_modER.pl”, and several VT score and transition matrices are provided as part of the Additional file 2.

We used the following versions of databases: Pfam v.27 [22], UniProt 2013_06 [46], PREFAB 4.0 [38], and SABmark 1.65 [39].

## Endnotes

^1^ There is an exception for a residue inserted after a deleted ancestral residue that makes the macroscopic TKF91 model not a linear model, strictly speaking.

^2^ We could propose that the substitution score also adds the contribution corresponding to $\log \mathrm {T}^{t}_{\text {MM}}$.

^3^ This does not mean that a dynamic programming algorithm for the AFR model at *t*=1 would be identical to that of the (-11/-1/BLOSUM62) Smith-Waterman algorithm. A one-to-one correspondence between the Smith-Waterman algorithm and a symmetric pair HMM is not possible, not even for a fixed-time parameterization. The reason is that while Smith-Waterman uses arbitrary scores, in a pair HMM each transition has to be accounted for as a probability. In addition to not being able to use one unique open score, another example of the lack of correspondence is in the score of a match. Smith-Waterman assumes that the score of a match is just the emission substitution score *S*(*a,b*); its probabilistic counterpart requires adding the contribution of the match-to-match transition, *S*(*a,b*)+logT_MM_. It does not appear that these two schemes can be reconciled with each other.

## Additional files

Additional file 1
**This supplement provides details about the sequence evolutionary models described in the manuscript, their differential equations and analytic solutions (when existing).** This supplement also collects our observations regarding previously existing evolutionary models, mainly tkf91 and tkf92. (PDF 972 kb)

Additional file 2
**Tarball that contains the source code for the programs introduced in this manuscript, as well as the Perl scripts used to generate the experiments in this work and the results of the experiments.** (ZIP 42,496 kb)

## References

[1] Altschul SF, Madden TL, Schaffer AA, Zhang J, Zhang Z, Miller W (1997). Gapped BLAST and PSI-BLAST: A new generation of protein database search programs. Nucl Acids Res.

[2] Eddy SR (1998). Profile hidden Markov models. Bioinformatics.

[3] Durbin R, Eddy SR, Krogh A, Mitchison GJ (1998). Biological Sequence Analysis: Probabilistic Models of Proteins and Nucleic Acids.

[4] Altschul SF (1993). A protein alignment scoring system sensitive at all evolutionary distances. J Mol Evol.

[5] Thorne JL, Kishino H, Felsenstein J (1991). An evolutionary model for maximum likelihood alignment of DNA sequences. J Mol Evol.

[6] Thorne JL, Kishino H, Felsenstein J (1992). Inching toward reality: an improved likelihood model of sequence evolution. J Mol Evol.

[7] Bishop MJ, Friday AE (1985). Evolutionary trees from nucleic acid and protein sequence. Proc R Soc B.

[8] Bishop MJ, Thompson EA (1986). Maximum likelihood alignment of DNA sequences. J Mol Biol.

[9] Metzler D, Fleissner D, Wakolbinger A, von Haeseler A (2001). Assessing variability by joint sampling of alignments and mutation rates. J Mol Evol.

[10] Bouchard-Côté A, Jordan MI. Evolutionary inference via the Poisson indel process. 2012. PNAS 10.1073/pnas.1220450110. 10.1073/pnas.1220450110 PMC3557041 23275296

[11] Mitchison GJ, Durbin RM (1995). Tree-based maximal likelihood substitution matrices and hidden Markov models. J Mol Evol.

[12] Mitchison GJ (1999). A probabilistic treatment of phylogeny and sequence alignment. J Mol Evol.

[13] Qian B, Goldstein RA (2003). Detecting distant homologs using phylogenetic tree-based HMMs. Proteins.

[14] McGuire AM, Hughes JD, Church GM (2000). Conservation of DNA regulatory motifs and discovery of new motifs in microbial genomes. Genome Res.

[15] Rivas E, Eddy SR (2008). Probabilistic phylogenetic inference with insertions and deletions. PLoS Comput Biol.

[16] Knudsen B, Miyamoto MM (2003). Sequence alignments and pair hidden Markov models using evolutionary history. J Mol Biol.

[17] Miklós I, Toroczkai Z, Gascuel O, Moret BME (2001). An improved model for statistical aligment. WABI 2001.

[18] Miklós I, Lunter GA, Holmes I (2004). A “Long Indel” model for evolutionary sequence alignment. Mol Biol Evol.

[19] Reese JT, Pearson WR (2002). Empirical determination of effective gap penalties for sequence comparison. Bioinformatics.

[20] Pearson WR (2000). Flexible sequence similarity searching with the FASTA3 program package. Meth Mol Biol.

[21] Eddy SR (2011). Accelerated profile HMM searches. PLoS Comp Biol.

[22] Finn RD, Clements J, Eddy SR (2011). HMMER web server: Interactive sequence similarity searching. Nucl Acids Res.

[23] Punta M, Coggill PC, Eberhardt RY, Mistry J, Tate J, Boursnell C (2012). The Pfam protein families database. NAR.

[24] Wheeler TJ, Clements J, Eddy SR, Hubley R, Jones TA, Jurka J (2013). Dfam: a database of repetitive DNA based on profile hidden Markov models. Nucl Acids Res.

[25] Eddy SR (2008). A probabilistic model of local sequence alignment that simplifies statistical significance estimation. PLoS Comput Biol.

[26] Karplus K (2009). SAM-T08, HMM-based protein structure prediction. Nucleic Acids Res.

[27] Gonzalez MW, Pearson WR (2010). Homologous over-extension: a challenge for iterative similarity searches. Nucl Acids Res.

[28] Mills LJ, Pearson WR (2013). Adjusting scoring matrices to correct overextended alignments. Bioinformatics.

[29] Lunter G (2007). Probabilistic whole-genome alignments reveal high indel rates in the human and mouse genomes. Bioinformatics.

[30] Wang J, Keightley PD, Johnson T (2006). MCALIGN2: Faster, accurate global pairwise alignment of non-coding DNA sequences based on explicit models of indel evolution. BMC Bioinformatic.

[31] Cartwright RA (2009). Problems and solutions for estimating indel rates and length distributions. Mol Biol Evol.

[32] Krogh A, Brown M, Mian IS, Sjölander K, Haussler D (1994). Hidden Markov models in computational biology: Applications to protein modeling. J Mol Biol.

[33] Hein J (2001). An algorithm for statistical alignment of sequences related by a binary tree. Pac Symp Biocomput.

[34] Smith TF, Waterman MS (1981). Identification of common molecular subsequences. J Mol Biol.

[35] Pearson WR (1995). Comparison of methods for searching protein sequence databases. Protein Sci.

[36] Pearson WR (2013). Selecting the right similarity-scoring matrix. Curr Protocol Bioinform.

[37] Rivas E (2005). Evolutionary models for insertions and deletions in a probabilistic modeling framework. BMC Bioinformatics.

[38] Edgar RC (2010). Quality measures for protein alignment benchmarks. Nucleic Acids Res.

[39] Van Walle I, Lasters I, Wyns L (2005). SABmark–a benchmark for sequence alingnment that covers the entire known fold space. Bioinformatics.

[40] van Rijsbergen CJ (1979). Information Retrival.

[41] Mirarab S, Warnow T (2011). FastSP: Linear time calculation of alignment accuracy. Bioinformatics.

[42] Camacho C, Coulouris G, Avagyan V, Ma N, Papadopoulos J, Bealer K (2009). BLAST+: Architecture and applications. BMC Bioinformatics.

[43] Liu Y, Schmidt B, Maskell DL (2010). MSAProbs: multiple sequence alignment based on pair hidden Markov models and partition function posterior probabilities. Bioinformatics.

[44] Edgar RC (2004). MUSCLE: a multiple sequence alignment method with reduced time and space complexity. BMC Bioinformatics.

[45] Müller T, Spang R, Vingron M (2002). A comparison of Dayhoff’s estimator, the resolvent approach and a maximum likelihood method. Mol Biol Evol.

[46] The UniProt Consortium (2015). UniProt: a hub for protein information. Nucl. Acids Res.

