# Supplementary material for: Parameterizing sequence alignment with an explicit evolutionary model
Source: BMC Bioinformatics. 2015 Dec 10;16:406. doi: 10.1186/s12859-015-0832-5 (PMC4676179; doi:10.1186/s12859-015-0832-5)
Supplement: Additional file 1 — This supplement provides details about the sequence evolutionary models described in the manuscript, their differential equations and analytic solutions (when existing). This supplement also collects our observations regarding previously existing evolutionary models, mainly tkf91 and tkf92. (PDF 972 kb) [file 12859_2015_832_MOESM1_ESM.pdf]

Parameterizing sequence alignment with an explicit evolutionary model

Elena Rivas & Sean R. Eddy*

Department of Molecular and Cellular Biology, Harvard University, Cambridge, MA 02138, USA
*Howard Hughes Medical Institute, Chevy Chase, MD 20815, USA

Supplemental Material

This supplement provides details about the sequence evolutionary models described in the manuscript, their differential equations and analytic solutions (when existing). This supplement also collects our observations regarding previously existing evolutionary models, mainly TKF91 and TKF92.

Evolutionary models compatible with standard affine gap cost

Obtaining a probabilistic model of sequence evolution requires finding a time dependent analytic description for the probability distribution of descendant sequences given an ancestral sequence $P_t(D \mid A)$ as a function of a finite number of parameters. The "time" is a continuous variable that reflects in arbitrary units the degree of divergence of a descendant sequence relative to its ancestor (with time zero meaning no divergence, and time infinity meaning evolved sequences are indistinguishable from having been sampled from a time independent stationary distribution of the model). The parameters of the model (usually referred to as rate constants for reasons that will become clear later) are time-independent non-negative values. A specific continuous-time analytic description of the probability distribution $P_t(D \mid A)$ as a function of its rate parameters is referred to as an evolutionary model, and because it describes the behavior for any arbitrary evolutionary distance (branch length), we refer to it as the *macroscopic* evolutionary model. The joint probability of an ancestral and descendant sequence $P_t(A, D)$ is simply the product of the conditional distribution $P_t(D \mid A)$ times the proposed distribution of ancestral sequences $P(A)$. In the case of pair HMMs, we assume for $P(A)$ an arbitrary time-independent geometric length distribution of parameter p, as is usually the constraint imposed by pair HMMs. For profile HMMs, the profile consensus plays the role of an ancestral sequence of fixed length, and we calculate the conditional probability $P_t(D \mid A)$ of the (descendant) sequence given the profile (ancestor).

In order to obtain the macroscopic model, we propose for $P_t(D \mid A)$ a suite of evolutionary events allowed at infinitesimal times. The parameters of these instantaneous events that define the behavior at very small times are referred to as the rate constants. We refer to the set of events allowed at infinitesimal time as the *microscopic* component of the evolutionary model.

Traditionally, the microscopic model is proposed first, from which the macroscopic model follows by solving the corresponding differential equations. Here, we take the opposite approach, as we try to identify the most realistic microscopic models compatible with the macroscopic architecture of a standard affine pair or profile HMM. Unfortunately, it is not always possible to associate a reasonable set of microscopic events with a desired macroscopic architecture.

Affine macroscopic model with ancestral memory

An immediate consequence of requiring that the evolutionary model maps to a standard profile HMM is that the probability distribution must allow the separation of the length-dependent terms (transition probabilities) from the actual residue values (emission probabilities). The evolution of residue substitutions has been well studied for proteins [1–6], as well as for DNA [7–12], and even for RNA basepairs [13]. Our models can adopt any standard residue substitution process. Therefore, from now on unless otherwise stated, the term $P_t(D \mid A)$ will refer only to the length-dependent part of the distribution (leaving aside the actual residue emission probabilities).

Here, we recall an important concept "**insert**" introduced already in the main manuscript. "Insert" specifically stands for the collection of all inserted residues in between any two ancestral positions, regardless of whether the associated ancestral residue is alive or not at the time.

An immediate consequence of requiring a map to a standard profile HMM is that we should be able to describe separately the fate of each ancestral residue. Profiles force a "memory" of ancestral residues, because profile match states are ancestral residues. For example, $\mathrm{M} \rightarrow (\mathrm{I})_n \rightarrow \mathrm{D} \rightarrow (\mathrm{I})_m \rightarrow M$ is two inserts, not one. The parameters controlling the $(\mathrm{I})_n$ insert are different from those controlling the $(\mathrm{I})_m$ insert, regardless of whether the intermediate ancestral position is present or not. Thus, we can break down the problem by expressing the probability $P_t(D \mid A)$ as a product of each individual ancestral residue

$$P_t(D \mid A = \{a_1 \dots a_L\}) = \prod_{i=1}^{L} P_t(\{d\}_i \mid a_i),$$

where $\{d\}_i$ is the collection of residues in the descendant sequence that are associated to an given ancestral residue a_i.

Another consequence of this ancestral memory is that the fate of an insert becomes independent of whether the associated ancestral position is alive or dead at a given time. Thus, we can write the probability of a given ancestral residue a as a function of a handful of elementary probability functions: $\gamma(t)$ (or γ_t for short) is the probability that an ancestral residue dies, and $P_n(t)$ (or P_n^t) is the probability of having an insert of length n after an ancestral residue regarding of whether the ancestral residue is dead or alive.

For a macroscopic evolutionary model with ancestral memory, the conditional probability that an ancestral residue a has survived at time t (and mutated to residue d), while producing an associated insert of length $n \geq 0$ is given by

$$P_t(\{d, i_i, \dots, i_n\} \mid a) = \begin{matrix} a & - & \dots & - \\ | & | & & | \\ d & i_1 & \dots & i_n \end{matrix} = \left[(1-\gamma_t)\ P_n^t\right]\ P_t(d \mid a)\, q_I(i_1) \dots q_I(i_n), \quad \text{for} \quad n \geq 0. \tag{1}$$

Here, $P_t(d \mid a)$ corresponds to any arbitrary substitution matrix. q_I is the emission distribution for inserted residues, which we assume to be time independent. The distribution q_I does not need to be same as that of ancestral residues (π) , nor do they need to match the saturation probabilities of the substitution matrix.

Consequently, the conditional probability that an ancestral residue has died at time t leaving an insert of length $n \geq 0$ is given by

$$P_t(\{i_i, \dots, i_n\} \mid a) = \begin{matrix} a & - & \dots & - \\ | & | & & | \\ - & i_1 & \dots & i_n \end{matrix} = \left[\gamma_t\, P_n^t\right]\ q_I(i_1) \dots q_I(i_n), \quad \text{for} \quad n \geq 0. \tag{2}$$

This macroscopic model admits a generalization of $\gamma(t)$ in which we can distinguish whether the ancestral residue occurs after another surviving residue (which we designate with M), after an ancestral residue that has disappeared (designated with D), after some residues have been inserted (I) or before any ancestral residue (B). All results presented here for $\gamma(t)$ generalize to $\gamma^{\{B,M,D,I\}}(t)$ as we will point out in different places. This generalization corresponds to transition probabilities from M (match), D (delete), I (insert) and B (begin) states of a profile or pair HMM.

In the type of Markov models that are typically used to describe sequence homology, the probability of an insert $P_n(t)$ admits only one of two forms: linear or affine. A linear gap cost assumption corresponds to a particular form for $P_n(t)$ given by

$$P_n^{\text{LINEAR}}(t) = (1-\beta_t)\,{\beta_t}^n \quad \text{for} \quad n \geq 0, \tag{3}$$

where β_t is a probability function, that corresponds to the cost of any inserted residue.

An affine gap cost assumption corresponds to the particular form for P_n^t,

$$P_n^{\text{AFFINE}}(t) = \begin{cases} (1-\beta_t) & \text{for} \quad n = 0, \\ \beta_t\,(1-\eta_t)\,{\eta_t}^{n-1} & \text{for} \quad n \geq 1, \end{cases} \tag{4}$$

where β_t and η_t are both probability functions. The probability of the first residue in an insert given by $\beta_t\,(1-\eta_t)$ is different from that of any other subsequent inserted residues, which is given by η_t. This is the case of interest to us because it has been adopted by most popular sequence alignment tools. An affine gap cost model fits under a three state HMM (see Figure 2). A linear model is a particular case of the affine model for $\beta_t = \eta_t$, and it can be described minimally with a one state HMM.

Next we investigate plausible microscopic evolutionary models and whether their macroscopic solution can fit into a standard (linear or affine) gap cost.

The microscopic Geometric (GM) model

We thought at first that a microscopic model that would allow single insertion and deletion events of geometrically distributed residues would result in a macroscopic model with geometrically distributed inserts (hence an affine cost). To that effect, we proposed the Geometric (GM) model which allows an arbitrary number of residues to appear or disappear in one single event according to geometric distributions.

The evolutionary events allowed by the microscopic GM model are,

infinitesimal time events for inserted residues

$(1-s_I)(s_I)^{n-1}\,\lambda\,\delta t$	Addition of a *new* insert of length n	
$(1-s_D)(s_D)^{n-1}\,\mu\,\delta t$	Deletion of a *whole* insert of length n	
$(1-v_I)(v_I)^{x-1}\,\lambda_I\,\delta t$	Addition of x residues into an existing insert	(5)
$(1-v_D)(v_D)^{x-1}\,\mu_I\,\delta t$	Deletion of x residues from an existing insert provided that the insert still exists after the deletion	

The rates μ, λ, μ_I, λ_I are positive constants, and s_I, s_D, v_D, v_I are constant Bernoulli probability parameters.

The microscopic GM model distinguishes whether the added residues "start" a new insert (with rate λ) or simply expand an existing one (with rate λ_I). It also distinguishes whether we are shrinking an

insert (with rate μ_I) or removing an insert all together (with rate μ). Notice that residues inserted in one single event do not have any memory of how they were created, and can be deleted at a later time independently from each other (that is, this is not a fragment model). The only memory that an inserted residue retains is that of the ancestral position it belongs to (the ancestral memory effect).

Regarding the fate of ancestral residues, the microscopic GM model is the simplest possible one, as we suspect it is the only one compatible with standard pair and profile HMMs, where ancestral residues are deleted individually. Thus, we propose that at infinitesimal times ancestral residues can be deleted one at a time with positive rate μ_A,

infinitesimal time events for ancestral residues

$$\mu_A\,\delta t \quad \text{Deletion of an ancestral residue} \tag{6}$$

Affine solution for deletions

The differential equation for the elementary probability function $\gamma(t)$ which corresponds to the probability at time t that an ancestral residue has died is given by

Fate of ancestral residues differential equation

$$\dot{\gamma}_t = \mu_A\,(1-\gamma_t), \tag{7}$$

with the initialization condition

$$\gamma(t=0) = 0.$$

That is, an alive residue at macroscopic time t, which has probability $(1-\gamma_t)$, can be deleted after an additional infinitesimal time δt with probability $\mu_A\,\delta t$.

The solution for the probability controlling the fate of an ancestral residue is given by

Fate of ancestral residues macroscopic solution

$$\gamma(t) = 1 - e^{-\mu_A\,t}. \tag{8}$$

In the early work of Bishop and Friday [14], $1-\gamma_t = e^{-\mu t}$ was coined the "DNA reliability" function, and μ was referred to as the "chance failure" rate. This function also appears in the TKF91 and TKF92 models.

A generalization can be added relative to Bishop's DNA reliability still consistent with a standard HMM by considering that ancestral residues could be deleted with different rates depending on their context. For the general solution, we only need to introduce different rates μ_A^{X} depending on whether the residue being deleted appears after a preceding "X" state which could be: a begin "B", Match "M", Delete "D" or Insert "I" state. This generalization allows us for instance to choose $\mu_A^{\mathrm{M}} < \mu_A^{\mathrm{D}}$, then the cost of initiating an ancestral deletion is higher than that of extending one, thus making the model affine

for ancestral residues. We anticipate that more realistic treatment of ancestral deletions will fall outside the realm of standard HMM formulations.

Geometrically distributed insertions do not produce geometrically distributed inserts

The proof for this statement is given later in the Section "The General (GM) model does not have an affine macroscopic solution". In that section, we first obtain the differential equations that a finite-time solution of the GM model should satisfy, and then we show that a solution with a geometric form cannot satisfy those differential equations. In fact, we do not know if a closed-form analytic (non-geometric) solution exists for the GM model.

Here, we test by numerical integration the macroscopic of the GM model, and we present examples of its non-geometric macroscopic behavior. Results are provided in Figure 1.

Figure 1A shows that for arbitrary values of the parameters geometrically distributed insertions do not produce geometrically distributed macroscopic inserts. Thus, the macroscopic version of the GM model does not correspond to a standard three-state HMM generally used in sequence alignment.

This result holds even for the simplified case (described in Figure 1B) where the microscopic parameters that control the addition/removal of whole inserts are the same as those controlling the addition/removal of residues to an existing inserts, that is,

$$\lambda = \lambda_I \qquad \mu = \mu_I$$
$$s_I = v_I \qquad s_D = v_D.$$

The result also holds when we give up geometric distributions for insertions: $s_I = s_D = v_I = v_D = 0$ (Figure 1C). The simple generalization that the rate of adding/removing the first/last residue that creates/destroys an insert is different from that of adding/removing one residue to an existing insert ($\lambda \neq \lambda_I$ and $\mu \neq \mu_I$) does not produced geometrically distributed inserts.

Only when both simplifications are taken together (Figure 1D), do we obtain geometrically distributed inserts. The model under those simplifications can be solved analytically, but it is linear (not affine) for insertions, as we describe next.

The AALI model: a particular solution affine for deletions

The AALI model is defined by the restriction that inserted residues are instantaneously added and removed one at the time, that is

$$s_I = s_D = v_I = v_D = 0,$$

as well as by the condition that the insertion/deletion rates are identical

$$\lambda_I = \lambda, \quad \mu_I = \mu.$$

The differential equations for the simplified AALI model are

AALI model differential equations ($\lambda_I = \lambda, \mu_I = \mu, s_I = s_D = v_D = v_I = 0$)

$$\begin{aligned} \dot{P}^t_{n\geq 0} = &+ \mu\,(n+1)P^t_{n+1} - \mu\,(n)\,P^t_n \qquad &(\mathbf{a}) \\ &+ \lambda\,(n)\,P^t_{n-1} \quad - \lambda(n+1)P^t_n, \qquad &(\mathbf{b}) \end{aligned} \tag{9}$$

with the initialization conditions

$$P_0(t=0) = 1, \quad P_n(t=0) = 0, \quad \text{for} \quad n > 0.$$

These differential equations consist of two types of terms: the left-hand side (positive) terms correspond to actual changes in the number of inserted residues from something else to n residues. The right-hand side (negative) terms correspond to changes that keep the number of inserted residues to be n. This is like how in a substitution rate matrix, the off-diagonal terms correspond to actual changes from one residue to a different one, while the negative terms correspond to the rate at which a residue remains unchanged. These terms play the role of an infinite-dimensioned rate matrix for the changes in the length of the inserted residues associated with a given ancestral residue.

The two off-diagonal terms that contribute after an infinitesimal δt to produce the end result of an insert of length n are:

(a) An insert of length $n+1$ could have lost one residue in one event with rate μ. This could have happened for any of the residues in the $n+1$ long insert.

(b) An insert of length $n-1$ could have added one residue in one event with rate λ. This could have happened at $n-1+1$ positions (including before the first residue in the insert) in the $n-1$ long insert.

Because $\sum_{n=0}^{\infty} P_n(t) = 1$ for all times, then it follows that $\sum_{n=0}^{\infty} \dot{P}_n(t) = 0$ also for all times. That is, as in a substitution rate matrix all changes for a given residue add up to zero, here, all changes for a given insert size also sum to zero.

To solve the AALI model, we propose a linear ansatz of the form,

$$P^{\text{AALI}}_{n\geq 0}(t) = (1-\beta^{\text{LI}}_t)\,(\beta^{\text{LI}}_t)^{\,n}, \quad \text{with} \quad \beta^{\text{LI}}(t=0) = 0. \tag{10}$$

Using the following equalities (derived from the above form),

$$\dot{P}^{\text{AALI}}_n(t) = \dot{\beta}^{\text{LI}}_t \left(\frac{n}{\beta^{\text{LI}}_t} - \frac{1}{1-\beta^{\text{LI}}_t}\right) P^{\text{AALI}}_n(t), \tag{11}$$

$$P^{\text{AALI}}_n(t) = \beta^{\text{LI}}_t\, P^{\text{AALI}}_{n-1}(t), \tag{12}$$

the differential equation becomes

AALI model differential equations for linear ansatz

$$\dot{\beta_t^{\rm LI}}\left(\frac{n}{\beta_t^{\rm LI}}-\frac{1}{1-\beta_t^{\rm LI}}\right) = +\,\mu\,(n+1)\beta_t^{\rm LI} - \mu\,(n) + \lambda\,(n)\frac{1}{\beta_t^{\rm LI}} - \lambda(n+1), \tag{13}$$

with the initialization condition

$$\beta^{\rm LI}(t=0)=0.$$

This equation can be rewritten as

$$\dot{\beta_t^{\rm LI}}\left(\frac{n}{\beta_t^{\rm LI}}-\frac{1}{1-\beta_t^{\rm LI}}\right) = (\lambda-\mu\,\beta_t^{\rm LI})\,(1-\beta_t^{\rm LI})\left(\frac{n}{\beta_t^{\rm LI}}-\frac{1}{1-\beta_t^{\rm LI}}\right). \tag{14}$$

Because the previous equation has to be true for all values of n, it requires

$$\dot{\beta_t^{\rm LI}} = (\lambda-\mu\,\beta_t^{\rm LI})\,(1-\beta_t^{\rm LI}). \tag{15}$$

The solution for this equation for the above initialization condition is

The macroscopic AALI model

$$\gamma^{\rm AALI}(t) = 1-e^{-\mu_A^{\{B,M,D,I\}}\,t}, \tag{16}$$

$$P_{n\geq 0}^{\rm AALI}(t) = (1-\beta_t^{\rm LI})\,(\beta_t^{\rm LI})^{\,n}, \tag{17}$$

$$\text{where}\quad \beta^{\rm LI}(t) = \begin{cases} \lambda\,\frac{1-e^{(\lambda-\mu)\,t}}{\mu-\lambda\,e^{(\lambda-\mu)\,t}} & \text{if} \quad \lambda\neq\mu, \\ \frac{\lambda\,t}{1+\lambda\,t} & \text{if} \quad \lambda=\mu. \end{cases} \tag{18}$$

A state machine representation of the AALI model is given in Figure 2. The AALI model is linear for insertions, but (technically) affine for deletions in the sense that the fate of ancestral residues is different depending on which state we were at previous to that ancestor.

The Linear (LI) model

When all rates of deleting ancestral residues are identical ($\mu_A^{\{B,M,D,I\}} = \mu_A$), we obtain the linear (LI) model. The LI model fits a standard one-state HMM (Figure S1). The LI model admits a particular solution that is reversible, the Linear Reversible (LR) model. We discuss this simpler model (and its connection to TKF91) next.

The Linear Reversible (LR) model: a particular case of the LI model

The LI model is in general not reversible for arbitrary values of the parameters. Reversibility is an unrealistic but convenient property of evolutionary models because then phylogenetic trees do not need

to be rooted, and in algorithms that establish a comparison between two extant sequences, either one of the sequences can be interpreted as the ancestor of the other one, which simplifies some algorithms. There is one particular combination of parameters that produce a reversible model (the LR model), which we derive in this section.

Starting from the LI model, reversibility requires that for the joint pair HMM in Figure S1(B), the role of the insert state "I" and the delete state "D" are interchangeable. That is, for the LI model to be reversible the transition probabilities of the joint pair HMM have to satisfy the conditions,

$$\mathrm{t_I}\, q_I(a) = \mathrm{t_D}\, p\, \pi(a). \tag{19}$$

This condition is satisfied (assuming $q_I = \pi$) by imposing the constraint,

$$\beta_t{}^{\mathrm{LR}} = p^{\mathrm{LR}}\, \gamma_t{}^{\mathrm{LR}}\, (1 - \beta_t{}^{\mathrm{LR}}). \tag{20}$$

That is, under a reversible model, inserting a residue, $P_t\binom{-}{a} = \beta_t{}^{\mathrm{LR}} \pi(a)$, is equivalent to creating and deleting an ancestral residue, $P_t\binom{a}{-} = p^{\mathrm{LR}}\, \pi(a)\, \gamma_t{}^{\mathrm{LR}}\, (1 - \beta_t{}^{\mathrm{LR}})$.

One can see using Eqs. (16,18), that this condition is satisfied for all divergence times if we select rate parameters satisfying,

$$\mu = \mu_A + \lambda, \tag{21}$$

and we assume that the geometric probability to generate ancestral residues instead of being a free parameter as in the LR model is given by

$$p^{\mathrm{LR}} = \frac{\lambda}{\mu_A}. \tag{22}$$

Since the equivalence needs to be true for all values of t, in particular for $t = \infty$, then we have

$$\frac{\lambda}{\mu} = \left(1 - \frac{\lambda}{\mu}\right) p^{\mathrm{LR}} \quad \text{for} \quad \lambda < \mu, \tag{23}$$

$$1 = 0 \times p^{\mathrm{LR}} \quad \text{for} \quad \lambda \geq \mu, \tag{24}$$

$$\tag{25}$$

which determines that the reversible model needs to satisfy $\lambda < \mu$, and the reversible stationary distribution should be

$$p^{\mathrm{LR}} = \frac{\lambda}{\mu - \lambda}. \tag{26}$$

Then, the reversibility condition in Eq. (20) becomes

$$\lambda \frac{1 - e^{(\lambda-\mu)\, t}}{\mu - \lambda\, e^{(\lambda-\mu)\, t}} = \frac{\mu - \lambda}{\mu - \lambda\, e^{(\lambda-\mu)\, t}} \left(1 - e^{-\mu_A\, t}\right) \frac{\lambda}{\mu - \lambda}. \tag{27}$$

That is,

$$1 - e^{(\lambda-\mu)\, t} = 1 - e^{-\mu_A\, t}. \tag{28}$$

which is true for all times under the condition $\mu - \lambda = \mu_A$.

Thus, the LR model is given by the particular values

The LR model

$$
\begin{aligned}
p^{\text{LR}} &= \frac{\lambda}{\mu_A}, \quad \text{with} \quad \lambda < \mu_A, \\
\gamma^{\text{LR}}(t) &= 1 - e^{-\mu_A\, t}, \\
P^{\text{LR}}_{n \geq 0}(t) &= (1 - \beta^{\text{LR}}_t)\,(\beta^{\text{LR}}_t)^{\,n}, \\
\text{where} \quad \beta^{\text{LR}}(t) &= \lambda\, \frac{1 - e^{-\mu_A\, t}}{(\mu_A + \lambda) - \lambda\, e^{-\mu_A\, t}}.
\end{aligned}
\tag{29}
$$

The linear state machine representation of the more general LI model (and its particular case the LR model) is given in Figure S1.

Comparison of the LR model to the TKF91 model

TKF91 is a necessary reference to any probabilistic model of indel evolution [15]. The LR model is the natural model to compare to TKF91, since they are both reversible. Before we do such a comparison, it is convenient to re-tell TKF91 using a formalism as close as possible to the one we have used to describe the LR model.

The microscopic TKF91 model

The TKF91 microscopic model is defined by,

TKF91 microscopic model

$$
\begin{aligned}
&\mu\, \delta t \quad \text{Deletion of a single ancestral residue.} \\
&\mu\, \delta t \quad \text{Deletion of a single inserted residue.} \\
&\lambda\, \delta t \quad \text{Addition of one single residue when associated to an existing residue.}
\end{aligned}
\tag{30}
$$

Under TKF91, ancestral residues can be deleted instantaneously and independently one at a time. Any inserted residue can also disappear instantaneously with the same rate as ancestral residues. This is one difference with the LR microscopic model which assumes that both deletion rates could be different. In non-position-specific models, TKF91's assumption of one single deletion rate μ makes sense since all residues are equivalent. In position-specific profile models, all deletion rates are going to be different anyway, so it is natural to allow the possibility that the deletion rates of ancestral versus inserted residues could be different as well.

Under TKF91's microscopic model, new insertions of one single residue can occur instantaneously associated either with an existing insertion or with a surviving ancestral residue (plus one more case before any ancestral residue). That is, if an ancestral residue is already dead at time t, no residue can appear associated with it (to its right by convention). Assuming that new residues can only appear associated to currently surviving residues is a reasonable assumption, and a crucial point that differentiates our LR

model from TKF91. The LR model makes the assumption that an insert can occur after any ancestral position, regardless or whether the corresponding ancestral residue is still alive or not. This assumption is reasonable within position-specific profile models, where the ancestral sequence is represented by the model consensus (collection of Match states) and those are always present.

TKF91's more realistic infinitesimal-time set of events is in turn responsible for the fact that there is a fundamental distinction between ancestral residues that die without any associated insert, versus those that die leaving at least one inserted residue (here we name them the "d_0" and "d_1" ancestral residues respectively). A d_1 ancestral residue can become a d_0 ancestor, but not the other way around. This distinction between dead ancestral residues is responsible for TKF91 not being exactly a linear gap cost model, which we discuss below.

All differences between our LR model and TKF91 can be traced back to the differences in their microscopic models.

The macroscopic TKF91 model

The macroscopic TKF91 model was originally defined by these probability functions [15]:

$[p_n^t]$ The probability that an ancestral residue survives with $n-1$ inserted residues associated to it.

$[q_n^t]$ The probability of an ancestral residue disappearing with n insertions associated to it (named p'_n in the original TKF91 paper [15]).

$[r_n^t]$ The probability of n residues being generated before any ancestral residue (named p''_n in the original paper).

These probabilities can be all expressed in terms of four elementary probability functions

$[p_1^t]$ The probability of a surviving ancestral residue.

$[q_0^t]$ The probability of an ancestral residue that disappears with no associated insertion.

$[q_1^t]$ The probability of an ancestral deletion accompanied by one residue insertion.

$[\beta_t^{\text{TKF}}]$ The probability of any additional insertion. That is, the probability of any inserted residue that is not associated with opening an insert after an ancestral deletion.

Not all four elementary probabilities are independent variables (in particular q_0^t ends up being proportional to β_t^{TKF}), but it is convenient to express them separately.

The relationships that TKF91 establishes are

$$
\begin{aligned}
p_n^t &= p_1^t\,(\beta_t^{\text{TKF}})^{n-1}, \quad \text{for } n \geq 1, & (31)\\
q_n^t &= q_1^t\,(\beta_t^{\text{TKF}})^{n-1}, \quad \text{for } n \geq 1, & (32)\\
q_0^t &= \frac{\mu}{\lambda}\,\beta_t^{\text{TKF}}, & (33)\\
r_n^t &= (1-\beta_t^{\text{TKF}})\,(\beta_t^{\text{TKF}})^{n}, \quad \text{for } n \geq 0. & (34)
\end{aligned}
$$

The time dependencies of the elementary functions are

$$\beta_t^{\text{TKF}} = \lambda \frac{1 - e^{(\lambda-\mu)\,t}}{\mu - \lambda\, e^{(\lambda-\mu)\,t}} \quad \xrightarrow{t\to\infty} \lambda/\mu, \quad (\text{for} \quad \lambda < \mu), \tag{35}$$

$$p_1^t = (1 - \beta_t^{\text{TKF}})\, e^{-\mu\, t} \quad \xrightarrow{t\to\infty} 0, \tag{36}$$

$$q_1^t = (1 - \beta_t^{\text{TKF}}) \left(1 - e^{-\mu\, t} - \frac{\mu}{\lambda}\, \beta_t^{\text{TKF}}\right) \quad \xrightarrow{t\to\infty} 0, \tag{37}$$

$$q_0^t = \frac{\mu}{\lambda}\, \beta_t^{\text{TKF}} \quad \xrightarrow{t\to\infty} 1. \tag{38}$$

TKF91 also introduces a geometric Bernoulli parameter p to generate ancestral residues, and a residue emission distribution $\pi(a)$ which is the same for ancestral residues and inserted residues. This residue distribution is usually assumed to be the equilibrium distribution of the substitution matrix used $P_t(a \mid b)$ which we leave unidentified as it is a constant variable in the comparison of the evolutionary models for insertions/deletions. Reversibility imposes that $p = \lambda/\mu$. We introduce the notation $p^{\text{TKF}}(a) = \frac{\lambda}{\mu}\pi(a)$ as the probability of generating ancestral residue 'a'.

TKF91 is not a linear gap cost model

The TKF91 model cannot be parameterized as a one-state linear HMM. The reason is that one needs to distinguish whether a deleted ancestral residue is followed by inserted residues (a D1 deletion) or not (a D0 deletion). The minimal HMM compatible with the TKF91 model has a S_1 state which allows insertions, and a S_0 state which forbids insertions. We represent simultaneously the pair and conditional versions of the HMM. In Figure S2(A), we present a minimal two-state HMM to represent the TKF91 model.

A point of confusion about TKF91 not being represented by a one-state HMM has arisen from the fact that there is a dynamic programming algorithm for TKF91 that does have a one-state Forward recursion representation [16]. However, it is important to notice that this one-state Forward recursion is not a one-state HMM. The transition parameters of this one-state recursion are not probabilities and they do not normalize. For instance, it is not possible to do a Viterbi optimal alignment algorithm or to sample evolutionary histories from the one-state recursion. The algorithm depends on a trick of rearranging dynamic programming cells, only valid for the Forward calculation, but not for Viterbi or sampling. We present such one state recursion in Figure S2(B), and provide its derivation next.

From the TKF91 two-state HMM one can write down the dynamic programming (DP) recursion for calculating the total probability of two sequences being related by any evolutionary history, the so called Forward algorithm. The naive Forward recursion uses two DP matrices, one for each state of the HMM in Figure S2(A), as follows

$$S_0(i,j) = p^{\text{TKF}}(x_i)\,\left[\,q_0^t\, S_0(i-1,j) + q_0^t\,(1-\beta_t^{\text{TKF}})\, S_1(i-1,j)\,\right] \qquad \text{D0 transitions} \tag{39}$$

$$S_1(i,j) = \begin{cases} +\,p^{\text{TKF}}(x_i)\, P_t(x_j \mid x_i) & \left[\frac{p_1^t}{1-\beta_t^{\text{TKF}}}\, S_0(i-1,j-1) + p_1^t\, S_1(i-1,j-1)\right] \quad \text{M transitions} \\ +\,p^{\text{TKF}}(x_i)\,\pi(x_j) & \left[\frac{q_1^t}{1-\beta_t^{\text{TKF}}}\, S_0(i-1,j-1) + q_1^t\, S_1(i-1,j-1)\right] \quad \text{D1 transitions} \\ +\,\pi(x_j) & \beta_t^{\text{TKF}}\, S_1(i,j-1) \quad \text{I transition} \end{cases} \tag{40}$$

The above recursion can be re-written in terms of just one DP matrix [17]. Here we obtain such one-state recursion after introducing

$$\hat{S}_0(i,j) = \frac{S_0(i,j)}{1-\beta_t^{\text{TKF}}},$$

which results in,

$$\hat{S}_0(i,j) = p^{\text{TKF}}(x_i)\, q_0^t\, \left[\hat{S}_0(i-1,j) + S_1(i-1,j)\right] \tag{41}$$

$$S_1(i,j) = \begin{cases} +\,p^{\text{TKF}}(x_i)\, P_t(x_j \mid x_i)\, p_1^t & \left[\hat{S}_0(i-1,j-1) + S_1(i-1,j-1)\right] \\ +\,p^{\text{TKF}}(x_i)\,\pi(x_j)\, q_1^t & \left[\hat{S}_0(i-1,j-1) + S_1(i-1,j-1)\right] \\ +\,\pi(x_j)\,\beta_t^{\text{TKF}} & S_1(i,j-1) \end{cases} \tag{42}$$

Introducing

$$X(i,j) = S_1(i,j) + \hat{S}_0(i,j),$$

the recursions for $\hat{S}_0$ and X are

$$\hat{S}_0(i,j) = p^{\text{TKF}}(x_i)\, q_0^t\; X(i-1,j) \tag{43}$$

$$X(i,j) = \begin{cases} +\,p^{\text{TKF}}(x_i)\, q_0^t & X(i-1,j) \\ +\,p^{\text{TKF}}(x_i)\, \left[P_t(x_j \mid x_i)\, p_1^t + \pi(x_j)\, q_1^t\,\right] & X(i-1,j-1) \\ +\,\pi(x_j)\,\beta_t^{\text{TKF}} & S_1(i,j-1) \end{cases} \tag{44}$$

Notice that one can rewrite $S_1(i,j-1)$ using the recursion for $\hat{S}_0$ as

$$\begin{aligned} S_1(i,j-1) &= X(i,j-1) - \hat{S}_0(i,j-1) \\ &= X(i,j-1) - p^{\text{TKF}}(x_i)\, q_0^t\, X(i-1,j-1). \end{aligned} \tag{45}$$

Finally, the recursion for X has the following form just depending on X itself,

$$X(i,j) = \begin{cases} +\,p^{\text{TKF}}(x_i)\, q_0^t & X(i-1,j) \\ +\,p^{\text{TKF}}(x_i)\, \left[P_t(x_j \mid x_i)\, p_1^t + \pi(x_j)\, q_1^t\, - \pi(x_j)\,\beta_t^{\text{TKF}}\, q_0^t\,\right] & X(i-1,j-1) \\ +\,\pi(x_j)\,\beta_t^{\text{TKF}} & X(i,j-1) \end{cases} \tag{46}$$

Notice that the $X(i-1, j-1)$ to $X(i, j)$ term in this recursion does not correspond to a match between an ancestral and a descendant position. In addition to a match, the term also includes an ancestral deletion followed by an insertion and also subtracts another term that does not correspond to a single evolutionary event.

Reversibility of TKF91 requires rearrangements in the alignments

Reversibility implies that in a given pairwise alignment of two extant sequences, there is a symmetry such that either extant sequence can take the ancestral or descendant sequence role resulting exactly with the same calculation. That is indeed the case of our LR model, but that simple inversion does not produce identical results for TKF91. For the TKF91 model, in addition to the inversion of sequences, one needs to alter the order of inserted residues precisely so that a descendant residue is always to the right of an ancestral one so that a d_1 deletion does not become a d_0 deletion in the reversed case. That is,

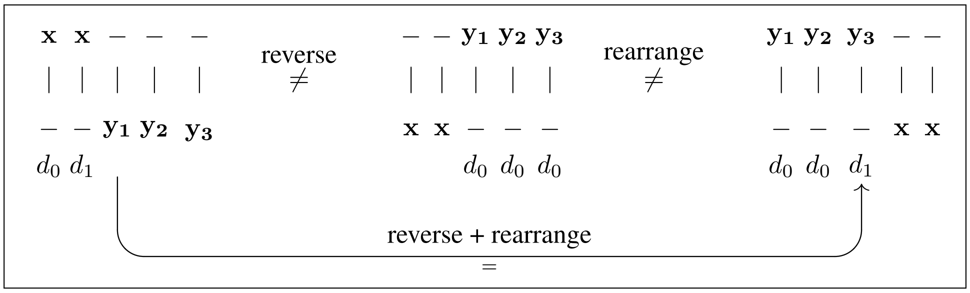

The probabilities of these alignments according to TKF91 are (omitting residue emissions)

$$
\begin{array}{ll}
(1-p^{\text{TKF}})\ (p^{\text{TKF}})^2\ (1-\beta_t^{\text{TKF}})^2\ (\gamma_{0t}^{\text{TKF}})^1\ (\beta_t^{\text{TKF}})^2\ (\gamma_t^{\text{TKF}}-\gamma_{0t}^{\text{TKF}}) & \text{original alignment,}\\
(1-p^{\text{TKF}})\ (p^{\text{TKF}})^3\ (1-\beta_t^{\text{TKF}})^1\ (\gamma_{0t}^{\text{TKF}})^3\ (\beta_t^{\text{TKF}})^2 & \text{reversed alignment,}\\
(1-p^{\text{TKF}})\ (p^{\text{TKF}})^3\ (1-\beta_t^{\text{TKF}})^2\ (\gamma_{0t}^{\text{TKF}})^2\ (\beta_t^{\text{TKF}})^1\ (\gamma_t^{\text{TKF}}-\gamma_{0t}^{\text{TKF}}) & \text{reversed+rearranged alignment.}
\end{array}
$$

with

$$
\begin{aligned}
\gamma_t^{\text{TKF}} &= 1-e^{-\mu t}\\
\gamma_{0t}^{\text{TKF}} &= \frac{\mu}{\lambda}\,\beta_t^{\text{TKF}}.
\end{aligned}
$$

It is easy to see that the original and reversed+rearranged alignments have the same probability by noticing that $p^{\text{TKF}}\,\gamma_{0t}^{\text{TKF}} = \beta_t^{\text{TKF}}$. There is not any other equivalence that would render the expression for the original alignment equal to that of the "reversed" alignment for all values of t.

This rearrangement is "evolutionarily" reasonable, but technically requires doctoring alignments so that such requirement is satisfied. For a pairwise alignment, it can be considered a convention to always rearrange to a delete/insert order instead of an insert/delete order. The problem comes with multiple alignment. Our linear LR model avoids such complications all together by avoiding the distinction between d_0 and d_1 deletions.

For the LR model all three alignments have the same probability given by

$$
\begin{aligned}
(1-p^{\text{LR}})\,p^{\text{LR}}\,(1-\beta_t{}^{\text{LR}})^2\,\gamma_t{}^{\text{LR}}\,(\beta_t{}^{\text{LR}})^n &= (1-p^{\text{LR}})\,(1-\beta_t{}^{\text{LR}})\,(p^{\text{LR}})^n\,(1-\beta_t{}^{\text{LR}})^n\,(\gamma_t{}^{\text{LR}})^n\,\beta_t{}^{\text{LR}}\\
&= (1-p^{\text{LR}})\,(1-\beta_t{}^{\text{LR}})\,(\beta_t{}^{\text{LR}})^{n+1}.
\end{aligned}
$$

This happens because the LR model satisfies the reversibility condition for a one-state joint pair HMM, that inserting a residue $\binom{-}{a}$ is equivalent to creating and deleting an ancestral residue $\binom{a}{-}$,

$$P\begin{pmatrix}-\\a\end{pmatrix} \equiv {\beta_t}^{\text{LR}}\,\pi(a) \;=\; p^{\text{LR}}\,\pi(a)\,{\gamma_t}^{\text{LR}}\,(1-{\beta_t}^{\text{LR}}) \equiv\; P\begin{pmatrix}a\\-\end{pmatrix}.$$

Affine evolutionary models using fragments à la TKF92

The AALI model imposes linear insert cost (bn) for an insert of length n. However, our aim is to allow affine cost (a+bn), such that the cost of adding a new insert (a+b) is usually much larger than the cost of adding one residue to an existing insert (b). A BLAST comparison using the typical (-11, -1) gap-open/gap-extend costs in 1/2 bit units (in conjunction with the BLOSUM62 substitution matrix) corresponds to $a = 0.30$ and $b = 0.79$; in PHMMER [18], $a = 0.02$ and $b = 0.40$.

There is a simple way of converting a macroscopic linear model into an affine model. This method was introduced with the conversion of TKF91 into TKF92 [19], and we give an interpretation of it in Figure 3A. It requires modifying a "linear" self-loop state (where the probability of entering the loop is the same as that of extending the loop) by adding another loop inside. One can see that the two loops can be merged together into a one self-looping state similar to the original, but now the loop extension probability is different from that of entering the loop.

For this state-machine manipulation to be compatible with a microscopic evolutionary model, one needs to invoke the concept of "fragments", such that all residues emitted between the "b" and "e" states in Figure 3A form an indivisible unit that can appears and disappears infinitesimally as a block of residues, but that cannot be broken apart by any subsequent evolutionary events. Also, the r parameter of the internal loop has to be time independent.

The TKF92 model [19] is such an "affine fragment" model, created by adding fragments to the TKF91 model [15]. The TKF92 model adds fragments not just to the Insert state, but also to the Match and Delete states [20]. Similarly, affine models can be created from the AALI model by adding fragments. That is the AFG model, described in Figure 5.

The affine fragment reversible (AFR) model compatible with sequence/sequence comparison algorithms

A particular case of the AFG model allows a symmetric description of insertions and deletions. It requires using (in Figure 3B) the linear reversible LR model as the underlying model, and adding fragments using the same geometric parameters for insertions and deletions, $r_I = r_D = r_X$. This model is named the affine fragment reversible (AFR) model, and it is described in Figure 4.

The insertion/deletion symmetry of the AFR model is due to the following property of the LR model,

$$\beta_t^{\text{LR}} = p^{\text{LR}}\,(1-\beta_t^{\text{LR}})\gamma_t^{\text{LR}}.$$

In detail,

AFR affine reversible model compatible with Smith-Waterman

$$
\begin{aligned}
\mathbf{T^t_{MM}} &= p^{\rm LR}\,(1-\beta^{\rm LR}_t)\,(1-\gamma^{\rm LR}_t)\,(1-r_M)+r_M \\
p^{\rm LR}\,(1-\beta^{\rm LR}_t)\,\gamma^{\rm LR}_t\,(1-r_M) = \mathbf{T^t_{MD}} &= \mathbf{T^t_{MI}} = \beta^{\rm LR}_t(1-r_M) \\
\mathbf{T^t_{ME}} &= (1-p^{\rm LR})\,(1-\beta^{\rm LR}_t)\,(1-r_M) \\
\mathbf{T^t_{DM}} &= \mathbf{T^t_{IM}} = p^{\rm LR}\,(1-\beta^{\rm LR}_t)\,(1-\gamma^{\rm LR}_t)\,(1-r_X) \\
p^{\rm LR}\,(1-\beta^{\rm LR}_t)\,\gamma^{\rm LR}_t\,(1-r_X)+r_X = \mathbf{T^t_{DD}} &= \mathbf{T^t_{II}} = \beta^{\rm LR}_t(1-r_X)+r_X \\
\beta^{\rm LR}_t(1-r_X) = \mathbf{T^t_{DI}} &= \mathbf{T^t_{ID}} = p^{\rm LR}\,(1-\beta^{\rm LR}_t)\,\gamma^{\rm LR}_t\,(1-r_X) \\
\mathbf{T^t_{DE}} &= \mathbf{T^t_{IE}} = (1-p^{\rm LR})\,(1-\beta^{\rm LR}_t)\,(1-r_X)
\end{aligned}
$$

In a more compact form, and using X ={I, D}

$$
\begin{aligned}
\mathbf{T^t_{MM}} &= \left(p^{\rm LR}\,(1-\beta^{\rm LR}_t)-\beta^{\rm LR}_t\right)\,(1-r_M)+r_M \\
\mathbf{T^t_{MX}} &= \beta^{\rm LR}_t(1-r_M) \\
\mathbf{T^t_{ME}} &= (1-p^{\rm LR})\,(1-\beta^{\rm LR}_t)\,(1-r_M) \\
\mathbf{T^t_{XM}} &= \left(p^{\rm LR}\,(1-\beta^{\rm LR}_t)-\beta^{\rm LR}_t\right)\,(1-r_X) \\
\mathbf{T^t_{XX}} &= \beta^{\rm LR}_t\,(1-r_X)+r_X \\
\mathbf{T^t_{XY}} &= \beta^{\rm LR}_t\,(1-r_X) \\
\mathbf{T^t_{XE}} &= (1-p^{\rm LR})\,(1-\beta^{\rm LR}_t)\,(1-r_X)
\end{aligned}
$$

After introducing $\beta^{\rm LR}_\infty = \frac{\lambda}{\lambda+\mu_A}$, we have

AFR affine reversible model compatible with Smith-Waterman

$$
\begin{aligned}
\mathbf{T^t_{MM}} &= \beta^{\rm LR}_\infty\,\frac{e^{-\mu_A\,t}}{1-\beta^{\rm LR}_\infty\,e^{-\mu_A\,t}}\,(1-r_M)+r_M \\
\mathbf{T^t_{MX}} &= \beta^{\rm LR}_\infty\,\frac{1-e^{-\mu_A\,t}}{1-\beta^{\rm LR}_\infty\,e^{-\mu_A\,t}}\,(1-r_M) \\
\mathbf{T^t_{ME}} &= \frac{1-2\,\beta^{\rm LR}_\infty}{1-\beta^{\rm LR}_\infty\,e^{-\mu_A\,t}}\,(1-r_M) \\
\mathbf{T^t_{XM}} &= \beta^{\rm LR}_\infty\,\frac{e^{-\mu_A\,t}}{1-\beta^{\rm LR}_\infty\,e^{-\mu_A\,t}}\,(1-r_X) \\
\mathbf{T^t_{XX}} &= \beta^{\rm LR}_\infty\,\frac{1-e^{-\mu_A\,t}}{1-\beta^{\rm LR}_\infty\,e^{-\mu_A\,t}}\,(1-r_X)+r_X \\
\mathbf{T^t_{XY}} &= \beta^{\rm LR}_\infty\,\frac{1-e^{-\mu_A\,t}}{1-\beta^{\rm LR}_\infty\,e^{-\mu_A\,t}}\,(1-r_X) \\
\mathbf{T^t_{XE}} &= \frac{1-2\,\beta^{\rm LR}_\infty}{1-\beta^{\rm LR}_\infty\,e^{-\mu_A\,t}}\,((1-r_X)
\end{aligned}
$$

The state-machine representation for the AFR model is given in Figure 4.

The affine insert fragment (AIF) model compatible with standard profile HMMs

The microscopic (evolutionary) interpretation of fragments requires that the indivisible fragments can appear and disappear in one single event. Inserted residues are amenable to such evolutionary events. The AIF model is a fragment model that adds fragments to inserted residues only. The AIF model is the only affine-fragment evolutionary model compatible with profile HMMs, since in profile HMMs only the state for inserted residues is self-looping.

Explicitly, the AIF model is given by

AIF affine model compatible with a profile HMM

$$P_n(t) = \begin{cases} 1-\beta_t & \text{for} \quad n=0. \\ \beta_t\,(1-\eta_t)(\eta_t)^{n-1} & \text{for} \quad n\geq 1. \end{cases} \tag{47}$$

$$\beta_t = \begin{cases} \lambda\,\frac{1-e^{(\lambda-\mu)\,t}}{\mu-\lambda\,e^{(\lambda-\mu)\,t}} & \text{for} \quad \lambda\neq\mu. \\ \frac{\lambda\,t}{1+\lambda\,t} & \text{for} \quad \lambda=\mu. \end{cases} \tag{48}$$

$$\eta_t = (1-r_I)\,\beta_t + r_I \tag{49}$$

The state-machine representation for the AIF model is given in Figure 5.

The General (GM) model does not have an affine macroscopic solution

The differential equations for the GM model

The differential equation for the generation of inserts relative to the ancestral sequence we have,

General Model (GM) differential equations

$$\dot{P}^t_0 = [\mathbf{a}] + \mu \sum_{m=1}^{\infty} (1 - s_D)\,(s_D)^{m-1}\, P^t_m \qquad - \lambda\, P^t_0, \tag{50}$$

$$\begin{aligned}\dot{P}^t_{n\geq 1} = [\mathbf{b}] &+ \lambda (1 - s_I)(s_I)^{n-1}\, P^t_0 && - \mu\,(1 - s_D)\,(s_D)^{n-1}\, P^t_n \\ [\mathbf{c}] &+ \mu_I\,(n+1) \sum_{m=1}^{\infty} (1 - v_D)(v_D)^{m-1}\, P^t_{n+m} && - \mu_I \sum_{m=1}^{n-1} (n - m + 1)\,(1 - v_D)(v_D)^{m-1}\, P^t_n \\ [\mathbf{d}] &+ \lambda_I \sum_{m=1}^{n-1} (n - m + 1)\,(1 - v_I)(v_I)^{m-1}\, P^t_{n-m} - \lambda_I\,(n+1) P^t_n, \end{aligned} \tag{51}$$

with the initialization conditions

$$P_0(t=0) = 1, \quad P_n(t=0) = 0, \quad \text{for} \quad n > 0.$$

These differential equations consist of two types of terms: the left-hand side (positive) terms corresponds to actual changes in the number of inserted residues from something else to n residues. The right-hand side (negative terms) corresponds to changes that keep the number of inserted residues to be n. In the same way that in a substitution rate matrix, the off-diagonal terms corresponds to actual changes from one residue to a different one, while the negative diagonal terms correspond to the rate at which a residue remains itself. These terms play the role of an infinite-dimensioned rate matrix for the changes in the length of the inserted residues associated with a given ancestral residue.

The explanation of the different off-diagonal terms that could contribute after an infinitesimal δt time to produce the end result of an insert of length n is as follows:

[a]: Any insert of arbitrary length (m) could have disappeared infinitesimally with rate $\mu\,(1-s_D)s_D{}^{m-1}$ for a final result of not having any inserted residues.

[b]: The whole n-length insert could have appeared infinitesimally with rate $\lambda\,(1 - s_I)s_I{}^{n-1}$.

[c]: An insert of length $n+m$ could have lost instantaneously m consecutive residues with rate $\mu_I\,(1 - v_D)v_D{}^{m-1}$. This could have happened at $n+1$ position in the $n+m$ long insert.

[d]: An insert of length $n-m$ (with $n-m \geq 1$, otherwise it would not be a pre-existing insert) could have added instantaneously m consecutive residues with rate $\lambda_I\,(1 - v_I)v_I{}^{m-1}$. This could have happened at $n-m+1$ position (including before the first residue in the insert) in the $n-m$ long insert.

Because $\sum_{n=0}^{\infty} P_n(t) = 1$ for all times, then it follows that $\sum_{n=0}^{\infty} \dot{P}_n(t) = 0$, also for all times. That is, as in a substitution rate matrix all changes for a given residue add up to zero, here, all changes for a given insert size, also sum to zero. Next, we show explicitly how that is the case. We also show a matrix formulation of the general solution for the GM model.

The condition $\sum_{n=0}^{\infty} P_n(t) = 1$ for all times, requires that $\sum_{n=0}^{\infty} \dot{P}_n(t) = 0$, also for all times. One can see by inspection that this condition on the time-derivatives is satisfied by the above differential equations. Spelling out all the terms for a few of the cases,

$$
\begin{aligned}
\dot{P}_0^t &= +\mu\,\left[(1-s_D)\,P_1^t + (1-s_D)s_D\,P_2^t + (1-s_D){s_D}^2\,P_3^t + \ldots\right] - \lambda\,P_0^t, \\
\\
\dot{P}_1^t &= +\lambda(1-s_I)\,P_0^t - \mu\,(1-s_D)\,P_1^t \\
&\quad +\mu_I\,(2)\,\left[(1-v_D)\,P_2^t + (1-v_D)v_D\,P_3^t + (1-v_D){v_D}^2\,P_4^t + \ldots\right] \\
&\quad -\lambda_I\,(2)\,P_1^t \\
\\
\dot{P}_2^t &= +\lambda(1-s_I)s_I\,P_0^t - \mu\,(1-s_D)\,s_D\,P_2^t \\
&\quad +\mu_I\,(3)\,\left[(1-v_D)\,P_3^t + (1-v_D)v_D\,P_4^t + \ldots\right] - \mu_I\,(2)\,(1-v_D)\,P_2^t \\
&\quad +\lambda_I\,(2)\,(1-v_I)\,P_1^t - \lambda_I\,(3)\,P_2^t \\
\\
\dot{P}_3^t &= +\lambda(1-s_I){s_I}^2\,P_0^t - \mu\,(1-s_D)\,{s_D}^2\,P_3^t \\
&\quad +\mu_I\,(4)\,\left[(1-v_D)\,P_4^t + \ldots\right] - \mu_I\,\left[(3)\,(1-v_D)\,P_3^t + (2)\,(1-v_D)v_D\,P_3^t\right] \\
&\quad +\lambda_I\,\left[(3)\,(1-v_I)\,P_2^t + (2)\,(1-v_I)v_I\,P_1^t\right] - \lambda_I\,(4)\,P_3^t \\
\\
\dot{P}_4^t &= +\lambda(1-s_I){s_I}^3\,P_0^t - \mu\,(1-s_D)\,{s_D}^3\,P_4^t \\
&\quad +\mu_I\,(5)\,\left[(1-v_D)\,P_5^t + \ldots\right] - \mu_I\,\left[(4)\,(1-v_D)\,P_4^t + (3)\,(1-v_D)\,v_D\,P_4^t + (2)\,(1-v_D){v_D}^2\,P_4^t\right] \\
&\quad +\lambda_I\,\left[(4)\,(1-v_I)\,P_3^t + (3)\,(1-v_I)\,v_I\,P_2^t + (2)\,(1-v_I){v_I}^2\,P_1^t\right] - \lambda_I\,(5)\,P_4^t \\
\\
\dot{P}_5^t &= \ldots
\end{aligned}
$$

For clarity, terms that cancel out are displayed in the same line (all terms in line 1 cancel out, all terms in line 2 cancel out...).

The differential equations above can be written in a matrix form

$$
\dot{P}_n^t = \sum_{m=0}^{\infty} M_{nm} P_m^t, \quad n \geq 0, \tag{52}
$$

where the infinite-dimensioned matrix M is given by,

$$M = \left(\begin{array}{cccccccc}
 & 0 & 1 & 2 & & n & \\
0 & -\lambda & \mu(1-s_D) & \mu(1-s_D)(s_D) & \dots & \mu(1-s_D)(s_D)^{n-1} & \dots \\
1 & \lambda(1-s_I)s_I & -\mu(1-s_D)-2\lambda_I & 2\mu_I(1-v_D) & \dots & 2\mu_I(1-v_D)(v_D)^{n-2} & \dots \\
2 & \lambda(1-s_I)(s_I)^2 & 2\lambda_I(1-v_I) & -\mu(1-s_D)(s_D)-3\lambda_I-2\mu_I(1-v_D) & \dots & 3\mu_I(1-v_D)(v_D)^{n-3} & \dots \\
 & \vdots & \vdots & \vdots & & \vdots & \\
n & \lambda(1-s_I)(s_I)^{n-1} & 2\lambda_I(1-v_I)(v_I)^{n-2} & 3\lambda_I(1-v_I)(v_I)^{n-3} & \dots & \begin{array}{c}-\mu(1-s_D)(s_D)^{n-1}-(n+1)\lambda_I \\ -\mu_I\sum_{m=1}^{n-1}(n-m+1)(1-v_D)(v_D)^{m-1}\end{array} & \dots \\
n+1 & \lambda(1-s_I)(s_I)^{n} & 2\lambda_I(1-v_I)(v_I)^{n-1} & 3\lambda_I(1-v_I)(v_I)^{n-2} & \dots & (n+1)\lambda_I(1-v_I) & \dots \\
n+2 & \lambda(1-s_I)(s_I)^{n+1} & 2\lambda_I(1-v_I)(v_I)^{n} & 3\lambda_I(1-v_I)(v_I)^{n-1} & \dots & (n+1)\lambda_I(1-v_I)v_I & \dots \\
 & \vdots & \vdots & \vdots & & \vdots &
\end{array}\right).$$

Notice that the sum of the terms in each column is zero.

The differential equation in (52) is conceptually the same differential equation for substitution of residues, but infinite-dimensioned in this case. One can see that the general solution is

$$P_n^t = \sum_m \left(e^{tM}\right)_{nm} P_m^{t=0}. \tag{53}$$

Using the initialization conditions $P_0^{t=0} = 1$ and $P_{n\geq 1}^{t=0} = 0$, then one has

$$P_n^t = \left(e^{tM}\right)_{n0}. \tag{54}$$

We have transformed the problem of solving the differential equations into exponentiating a matrix.

An affine solution to the GM model?

An affine macroscopic solution would take the form

$$P_0^{\text{AFF}}(t) = (1-\beta_t^{\text{AFF}}) \tag{55}$$

$$P_{n\geq 1}^{\text{AFF}}(t) = \beta_t^{\text{AFF}}\,(1-\eta_t)\,{\eta_t}^{n-1}. \tag{56}$$

Notice that $\sum_{n=1}^{\infty} P_n^{\text{AFF}}(t) = \beta_t^{\text{AFF}} = 1 - P_0^{\text{AFF}}(t)$ as it should be.

In order to see if this ansatz form is compatible with the differential equations Eqs. (50,51), consider the particular simpler case in which inserts as well as inserted residues into inserts are added instantaneously one at the time, that is,

$$s_I = v_I = s_D = r_D = 0. \tag{57}$$

The differential equations for the GM model in Eqs. (50,51) simplify to

Particular model: $\lambda \neq \lambda_I, \mu \neq \mu_I, s_I = v_I = s_D = r_D = 0$

$$\dot{P}_0^t = +\mu P_1^t - \lambda P_0^t, \tag{58}$$

$$\begin{aligned} \dot{P}_1^t &= +\lambda P_0^t - \mu P_1^t \\ &+ \mu_I (2) P_2^t - \lambda_I (2) P_1^t, \end{aligned} \tag{59}$$

$$\begin{aligned} \dot{P}_{n\geq 2}^t &= +\mu_I (n+1) P_{n+1}^t - \mu_I (n) P_n^t \\ &+ \lambda_I (n) P_{n-1}^t - \lambda_I (n+1) P_n^t, \end{aligned} \tag{60}$$

with the initialization conditions

$$P_0(t=0) = 1, \quad P_n(t=0) = 0, \quad \text{for} \quad n > 0.$$

Using the following equalities (which follow from the affine ansatz form),

$$\dot{P}_{n\geq 1}^{\mathrm{AFF}}(t) = \left(\frac{\dot{\beta}_t^{\mathrm{AFF}}}{\beta_t^{\mathrm{AFF}}} - \frac{\dot{\eta}_t}{1-\eta_t} + (n-1)\frac{\dot{\eta}_t}{\eta_t} \right) P_n^{\mathrm{AFF}}(t) \tag{61}$$

$$P_{n\geq 2}^{\mathrm{AFF}}(t) = \eta_t P_{n-1}^{\mathrm{AFF}}(t) \tag{62}$$

we can rewrite the above equations under the affine ansatz as,

Affine ansatz for particular case: $\lambda \neq \lambda_I, \mu \neq \mu_I, s_I = v_I = s_D = r_D = 0$

$$\dot{\beta}_t^{\mathrm{AFF}} = -\mu \beta_t^{\mathrm{AFF}} (1-\eta_t) + \lambda (1 - \beta_t^{\mathrm{AFF}}), \tag{63}$$

$$\begin{aligned} \frac{\dot{\beta}_t^{\mathrm{AFF}}}{\beta_t^{\mathrm{AFF}}} - \frac{\dot{\eta}_t}{1-\eta_t} &= +\lambda \frac{1-\beta_t^{\mathrm{AFF}}}{\beta_t^{\mathrm{AFF}}(1-\eta_t)} - \mu \\ &+ \mu_I (2) \eta_t - \lambda_I (2), \end{aligned} \tag{64}$$

$$\begin{aligned} \frac{\dot{\beta}_t^{\mathrm{AFF}}}{\beta_t^{\mathrm{AFF}}} - \frac{\dot{\eta}_t}{1-\eta_t} + (n-1)\frac{\dot{\eta}_t}{\eta_t} &= +\mu_I (n+1) \eta_t - \mu_I (n) \\ &+ \lambda_I (n) \frac{1}{\eta_t} - \lambda_I (n+1) \end{aligned} \tag{65}$$

with the initialization conditions

$$\beta^{\mathrm{AFF}}(t=0) = 0, \quad \eta(t=0) = \eta_0 \quad 0 \leq \eta_0 < 1.$$

Introducing Eq. (64) into Eq. (65) and using the relationship

$$\mu_I (n+1) \eta_t - \mu_I (n) + \lambda_I (n) \frac{1}{\eta_t} - \lambda_I (n+1) = (\lambda_I - \mu_I \eta_t)(1-\eta_t) \left(\frac{n}{\eta_t} - \frac{1}{1-\eta_t} \right),$$

the system reduces to two differential equations

$$\dot{\beta_t^{\rm AFF}} = \lambda\,(1-\beta_t^{\rm AFF}) - \mu\,\beta_t^{\rm AFF}\,(1-\eta_t), \tag{66}$$

$$(n-1)\frac{\dot{\eta_t}}{\eta_t} = (\lambda_I - \mu_I\,\eta_t)\,(1-\eta_t)\left(\frac{n}{\eta_t}+\frac{1}{1-\eta_t}\right) + \mu - \lambda\,\frac{1-\beta_t^{\rm AFF}}{\beta_t^{\rm AFF}(1-\eta_t)} \tag{67}$$

For Eq. (67) to be consistent, and since the solution has to be valid for all values on n, the terms in n and the terms independent of n should give the same results. Unfortunately that is not the case. The terms in n produce a differential equation for η_t that is independent of μ and λ, while the equation that comes out from the terms independent of n (as well as the first equation from $\beta_t^{\rm AFF}$) both require a dependency of η_t on the rates μ and λ for adding/removing whole inserts,

$$\dot{\beta_t^{\rm AFF}} = \lambda\,(1-\beta_t^{\rm AFF}) - \mu\,\beta_t^{\rm AFF}\,(1-\eta_t), \tag{68}$$

$$\dot{\eta_t} = (\lambda_I - \mu_I\,\eta_t)\,(1-\eta_t), \quad \text{from the n dependent terms.} \tag{69}$$

$$\dot{\eta_t} = \left(\lambda\,\frac{(1-\beta_t^{\rm AFF})\eta_t}{\beta_t^{\rm AFF}(1-\eta_t)} - \mu\,\eta_t\right) - (\lambda_I - \mu_I\,\eta_t)\,\eta_t, \quad \text{from the n independent terms.} \tag{70}$$

These three equations can only be made consistent with each other under the additional assumptions

$$\eta_t = \beta_t^{\rm AFF}, \quad \lambda = \lambda_I, \quad \mu = \mu_I,$$

in which case we recover the LI model introduced in the previous section.

There is a simplified model that produces a macroscopic affine model. Consider the special conditions:

$$\mu = \mu_I = \lambda_I = 0, \tag{71}$$

which correspond to the rather unrealistic assumption that inserts can only be created as a whole, and cannot be deleted. The differential equations in this case become

Particular GM affine case $\mu = \lambda_I = \mu_I = 0$

$$\dot{P}_0^t = -\,\lambda\,P_0^t \tag{72}$$

$$\dot{P}_{n\geq 1}^t = +\,\lambda(1-s_I)(s_I)^{n-1}\,P_0^t \tag{73}$$

with the initialization conditions

$$P_n(t=0) = 0, \quad \text{for} \quad n \geq 1.$$

There is a simple affine solution given by

Particular GM **affine case** $\mu = \lambda_I = \mu_I = 0$

$$P_0^t = e^{-\lambda t} \tag{74}$$

$$P_{n\geq 1}^t = \left(1 - e^{-\lambda t}\right) (1 - s_I)(s_I)^{n-1}. \tag{75}$$

For other arbitrary values of parameters of the GM model, we have tested by simulation of the microscopic model that the corresponding macroscopic model produce inserts that do not follow a geometric distribution (Figure 1).

An alternative geometric affine (AGA) evolutionary model compatible with profile HMMs

We have already described the AIF fragment model as an affine evolutionary model compatible with profile HMMs. Here, we introduce another model, the alternative geometric affine (AGA) evolutionary model, also compatible with profile HMMs. The AIF model is a fragment model. The AGA model uses a different approach, not necessarily more realistic in its microscopic description, in which inserts (the whole collection of inserted residues between two ancestral positions) can appear or disappear as a whole in one single event, but they cannot grow or shrink. In the AIF model, a given insert can grow in one single event by adding a new fragment or shrink by removing an existing fragment. The state machine representation of the macroscopic versions of both models is given in Figure 5.

The AGA microscopic model

The AGA microscopic model allows for an insert of length n to appear with rate $(1 - s_I)(s_I)^{n-1}\,\lambda$ in between two ancestral positions, and an insert can be deleted in one single event with rate μ. The single events associated to the fate of an ancestral residue is the same as for the GM model, and we omit them here.

The microscopic AGA model for the generation of insertions is as follows,

infinitesimal time events for inserted residues

$(1 - s_I)(s_I)^{n-1}\,\lambda\,\delta t$	Addition of a *new* insert of length n
$\mu\,\delta t$	Deletion of a *whole* insert of any length

(76)

The rates μ, λ are positive constants, and s_I is a constant Bernoulli probability parameter. While in the AGA model all inserts have the same probability of being removed at infinitesimal time, in the GM model, inserts disappear according to a geometric distribution.

The AGA macroscopic model

The differential equations resulting from the above microscopic model are,

Alternative Geometric Affine (AGA) model differential equations

$$\dot{P}_0^t = +\mu \sum_{m=1}^{\infty} P_m^t \qquad - \lambda P_0^t, \qquad (a)$$

$$\dot{P}_{n\geq 1}^t = +\lambda(1-s_I)(s_I)^{n-1} P_0^t - \mu P_n^t \qquad (b)$$

with the initialization conditions

$$P_0(t=0) = 1, \quad P_n(t=0) = 0, \quad \text{for} \quad n \geq 1.$$

This is a very simple evolutionary process in which the interpretation of the off-diagonal (positive) terms is as follows,

(a) An empty insert (P_0^t) could be the result of an insert of arbitrary length (m) disappearing in one single event with rate μ.

(b) A whole n-length insert ($P_{n\geq 1}^t$) could have appeared in a single event with rate $\lambda(1-s_I)(s_I)^{n-1}$.

The diagonal (negative) terms are such that the condition $\sum_{n=0}^{\infty} \dot{P}_n(t) = 0$ is satisfied at all times.

The solution to these equations is given by

The AGA model compatible with a profile HMM

$$\beta_t = \frac{\lambda}{\lambda+\mu}\left(1 - e^{-(\lambda+\mu)\,t}\right) \qquad (77)$$

$$P_0^t = 1 - \beta_t \qquad (78)$$

$$P_{n\geq 1}^t = \beta_t\,(1-s_I)(s_I)^{n-1}. \qquad (79)$$

This model implies that for an ancestral sequence of length l, the expected number of inserts is $(l+1)\,\beta_t$, and the expected length of an insert is $1/(1-s_I)$.

References

1. Dayhoff MO, Schwartz RM, Orcutt BC (1978) A model of evolutionary change in proteins. In: Dayhoff MO, editor, Atlas of Protein Sequence and Structure, Washington DC: National Biomedical Research Foundation. pp. 345-352.

2. Henikoff S, Henikoff JG (1992) Amino acid substitution matrices from protein blocks. Proc Natl Acad Sci USA 89: 10915-10919.

3. Adachi J, Hasegawa M (1996) Model of amino acid substitution in proteins encoded by mitochondrial DNA. J Mol Evol 42: 459-468.

4. Müller T, Vingron M (2000) Modeling amino acid replacement. J Comput Biol 7: 761–776.

5. Whelan S, Goldman N (2001) A general empirical model of protein evolution derived from multiple protein families using a maximum-likelihood approach. Mol Biol Evol 18: 691–699.

6. Le SQ, Gascuel O (2008) An improved general amino acid replacement matrix. Mol Biol Evol 25: 1307–1320.

7. Jukes TH, Cantor C (1965) Evolution of protein molecules. In: Mammalian Protein Metabolism. Academic Press, pp. pp. 21-132.

8. Kimura M (1980) A simple method for estimating evolutionary rates of base substitutions through comparative studies of nucleotide sequences. J Mol Evol 16: 111-120.

9. Hasegawa M, Kishino H, Yano T (1985) Dating of the human-ape splitting by a molecular clock of mitochondrial DNA. J Mol Evol 22: 160–174.

10. Churchill GA (1989) Stochastic models for heterogeneous DNA sequences. Bull Math Biol 51: 79–94.

11. Yang Z (1994) Maximum likelihood phylogenetic estimation from DNA sequences with variable rates over sites: Approximate methods. J Mol Evol 39: 306–314.

12. Felsenstein J, Churchill G (1996) A hidden Markov model approach to variation among sites in rate of evolution. Mol Bio Evol 13: 93-104.

13. Muse SV (1995) Evolutionary analyses of DNA sequences subject to constraints on secondary structure. Genetics 139: 1429–1439.

14. Bishop MJ, Friday AE (1985) Evolutionary trees from nucleic acid and protein sequence. Proc R Soc B 226: 271-302.

15. Thorne JL, Kishino H, Felsenstein J (1991) An evolutionary model for maximum likelihood alignment of DNA sequences. J Mol Evol 33: 114-124.

16. Hein J (2001) An algorithm for statistical alignment of sequences related by a binary tree. Pac Symp Biocomput 6: 179-190.

17. Hein J, Wiuf C, Knudsen B, Moller MB, Wibling G (2000) Statistical alignment: computational properties, homology testing and goodness-of-fit. J Mol Biol 302: 265-279.

18. Eddy SR (2011) Accelerated profile HMM searches. PLoS Comp Biol 7: e1002195.

19. Thorne JL, Kishino H, Felsenstein J (1992) Inching toward reality: an improved likelihood model of sequence evolution. J Mol Evol 34: 3-16.

20. Lunter G, Drummond A, Miklós I, Hein J (2005) Statistical alignment: Recent progress, new applications, and challenges. Stat Meth in Mol Evol Nielsen, Rasmus (Ed.) Springer Verlag.

Supplemental Figures

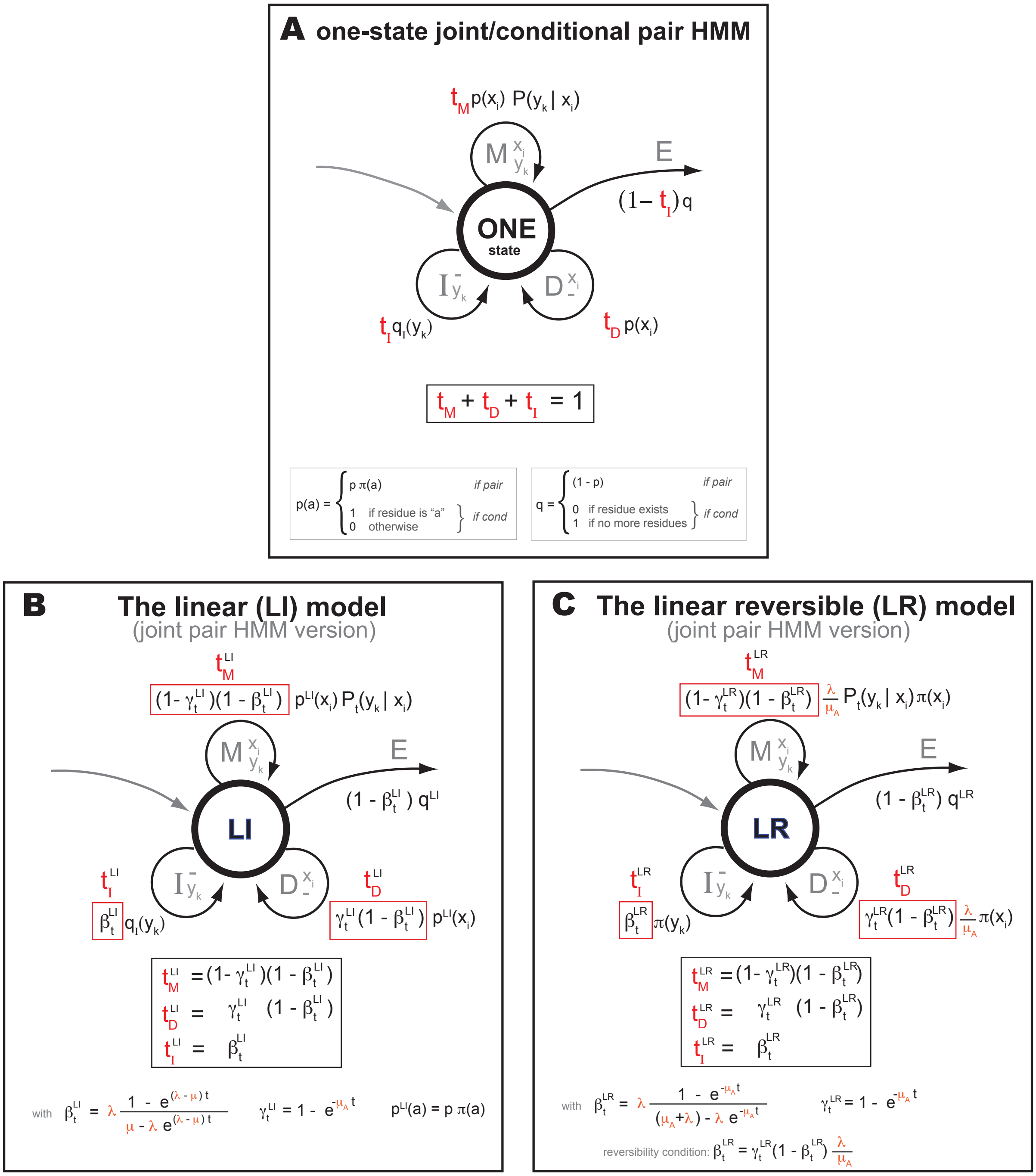
A one-state joint/conditional pair HMM
t_M p(x_i) P(y_k | x_i)
M
E
(1– t_I) q
ONE
state
I
D
t_I q_I(y_k)
t_D p(x_i)
t_M + t_D + t_I = 1
p(a) = p π(a) if pair; 1 if residue is "a", 0 otherwise if cond
q = (1 - p) if pair; 0 if residue exists, 1 if no more residues if cond
B The linear (LI) model
(joint pair HMM version)
t_M^LI
(1- γ_t^LI)(1 - β_t^LI) p^LI(x_i) P_t(y_k | x_i)
M
E
(1 - β_t^LI) q^LI
LI
I
D
t_I^LI
β_t^LI q_I(y_k)
t_D^LI
γ_t^LI(1 - β_t^LI) p^LI(x_i)
t_M^LI = (1- γ_t^LI)(1 - β_t^LI)
t_D^LI = γ_t^LI (1 - β_t^LI)
t_I^LI = β_t^LI
with β_t^LI = λ (1 - e^{(λ - μ) t}) / (μ - λ e^{(λ - μ) t})
γ_t^LI = 1 - e^{-μ_A t}
p^LI(a) = p π(a)
C The linear reversible (LR) model
(joint pair HMM version)
t_M^LR
(1- γ_t^LR)(1 - β_t^LR) λ/μ_A P_t(y_k | x_i) π(x_i)
M
E
(1 - β_t^LR) q^LR
LR
I
D
t_I^LR
β_t^LR π(y_k)
t_D^LR
γ_t^LR(1 - β_t^LR) λ/μ_A π(x_i)
t_M^LR = (1- γ_t^LR)(1 - β_t^LR)
t_D^LR = γ_t^LR (1 - β_t^LR)
t_I^LR = β_t^LR
with β_t^LR = λ (1 - e^{-μ_A t}) / ((μ_A+λ) - λ e^{-μ_A t})
γ_t^LR = 1 - e^{-μ_A t}
reversibility condition: β_t^LR = γ_t^LR(1 - β_t^LR) λ/μ_A

Figure S1. (A) A one-state (linear) joint/conditional pair HMM. Probabilistic version of the standard one-state recursion to align sequences under a linear gap cost. Here, we present simultaneously the joint version of the pair HMM (two sequences are simultaneously generated by the model) as well as the conditional version (one sequence is generated from a given fixed sequence). For the joint version, we assume one of the sequences is generated according to a geometric distribution of Bernoulli parameter p, and probability distribution π. Joint residue match emissions occur with probability $P_t(a,b) = \pi(a) P_t(b \mid a)$, and conditional match emissions occur with probability $P_t(b \mid a)$. Inserted residues follow a probability distribution q_I which in principle could be different from π. The transition parameters $\mathrm{t_M}, \mathrm{t_D}, \mathrm{t_I}$ are probabilities valued between zero and one. The HMM is normalized in its two versions as long as the transition probabilities satisfy the condition $\mathrm{t_M} + \mathrm{t_D} + \mathrm{t_I} = 1$. **(B) Standard one-state HMM parameterized by the Linear Insert (LI) evolutionary model.** We write the transition probabilities of the HMM in terms of two elementary probability functions γ_t (the probability of deleting an ancestral residue), and β_t (the probability of inserting a residue) such that the HMM is normalized for arbitrary probability functions γ_t and β_t. That is, $\mathrm{t_M} = (1-\beta_t)(1-\gamma_t)$, $\mathrm{t_D} = (1-\beta_t)\gamma_t$ and $\mathrm{t_I} = \beta_t$. Under the LI model, those elementary functions have time dependencies dictated by the microscopic model. The LI evolutionary model depends on three rate parameters: μ_A the rate of deleting an ancestral residue and μ, λ the rates of deleting/inserting individual residues previously inserted. The elementary functions depend on the microscopic rates as given in the figure. **(C) The reversible LR particular case.** In the LI model, the Bernoulli parameter p is another free parameter of the model. Under the restrictions: $\mu = \lambda + \mu_A$, $p = \lambda / \mu_A$ and $q_I = \pi$, the LI model becomes reversible, named the Linear Reversible (LR) model. For the LR model, we have the property that $\mathrm{t_I}^{\mathrm{LR}}\, q_I(a) = \mathrm{t_D}^{\mathrm{LR}}\, p^{\mathrm{LR}}(a)$, which results in a symmetry between the I and D states of the reversible joint pair HMM.

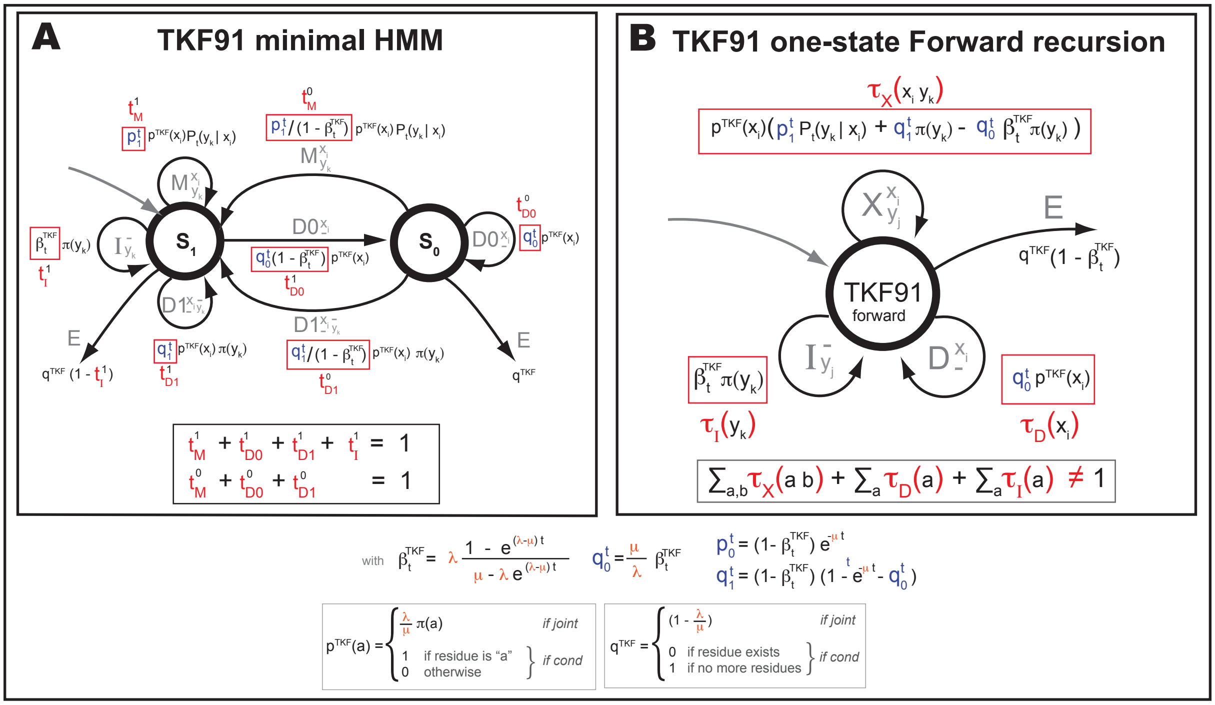

Figure S2. (A) Minimal (two-state) HMM representation of the TKF91 evolutionary model. The TKF91 model cannot parameterize a one-state linear HMM. The reason is that it needs to distinguish whether a deleted ancestral residue is followed by inserted residues (a D1 deletion) or not (a D0 deletion). The minimal HMM compatible with the TKF91 model has an S_1 state that allows subsequent insertions, and a S_0 state that forbids subsequent insertions. As in the previous cases, we represent simultaneously the pair and conditional versions of the HMM. We provide the conditions for the transition probabilities under which this two-state HMM is normalized (both in its pair or conditional version). Those normalization conditions are satisfied by the TKF91 macroscopic parameterization given in the figure. **(B) Non-probabilistic one-state recursion for the TKF91 Forward algorithm.** From the TKF91 two-state HMM, one can write down a dynamic programming (DP) recursion for calculating the total probability of two sequences being related by any evolutionary history, the so-called Forward algorithm. The naive Forward recursion uses two DP matrices, one for each state of the HMM. The Forward recursion for the TKF91 model can re-written in terms of just one DP matrix [17]. This figure describes the one-state Forward DP recursion for TKF91. It is important to notice that this recursion is not a one-state HMM. The transition parameters of this recursion are not probabilities and they do not normalize. The $X_{y_j}^{x_i}$ transition is not a match between positions x_i and y_j.
